# Sincast: a computational framework to predict cell identities in single-cell transcriptomes using bulk atlases as references

**DOI:** 10.1093/bib/bbac088

**Published:** 2022-03-31

**Authors:** Yidi Deng, Jarny Choi, Kim-Anh Lê Cao

**Affiliations:** Melbourne Integrative Genomics, School of Mathematics and Statistics, The University of Melbourne, Parkville, 3010, VIC, Australia; Centre for Stem Cell Systems, School of Biomedical Sciences, The University of Melbourne, Parkville, 3010, VIC, Country; Melbourne Integrative Genomics, School of Mathematics and Statistics, The University of Melbourne, Parkville, 3010, VIC, Australia; Melbourne Integrative Genomics, School of Mathematics and Statistics, The University of Melbourne, Parkville, 3010, VIC, Australia

**Keywords:** scRNA-seq, RNA-seq, pseudo-bulk, imputation, cell identity prediction

## Abstract

Characterizing the molecular identity of a cell is an essential step in single-cell RNA sequencing (scRNA-seq) data analysis. Numerous tools exist for predicting cell identity using single-cell reference atlases. However, many challenges remain, including correcting for inherent batch effects between reference and query data andinsufficient phenotype data from the reference. One solution is to project single-cell data onto established bulk reference atlases to leverage their rich phenotype information. Sincast is a computational framework to query scRNA-seq data by projection onto bulk reference atlases. Prior to projection, single-cell data are transformed to be directly comparable to bulk data, either with pseudo-bulk aggregation or graph-based imputation to address sparse single-cell expression profiles. Sincast avoids batch effect correction, and cell identity is predicted along a continuum to highlight new cell states not found in the reference atlas. In several case study scenarios, we show that Sincast projects single cells into the correct biological niches in the expression space of the bulk reference atlas. We demonstrate the effectiveness of our imputation approach that was specifically developed for querying scRNA-seq data based on bulk reference atlases. We show that Sincast is an efficient and powerful tool for single-cell profiling that will facilitate downstream analysis of scRNA-seq data.

## Introduction

Single-cell RNA sequencing (scRNA-seq) allows for the study of cell-specific variations in transcriptional states at an unprecedented resolution. One essential step in scRNA-seq data analysis is to characterize cell molecular identity, either *de novo* or with existing vocabularies of known cell types or states. Numerous computational tools have been developed for predicting cell identity using other single-cell atlases as references [3, 4]. However many challenges remain, including integrating atlases from independent studies to build comprehensive atlases that are generalizable, annotating reference cells accurately and tuning the parameters of these prediction tools appropriately [5]. Furthermore, the reference and query data effectively represent separate batches. Correcting for batch effects is required before direct comparisons can be made. Using data integration to address this issue is difficult from both a statistical and data analysis perspective [6, 7]. During the reference-query integration task, biological and batch effects are confounded, resulting in the potential removal of large amount of biological variation that is considered as batch variation.

In light of these challenges, bulk sequencing data represent a valuable resource for building reference atlases, as the samples can be of high quality, well replicated and well annotated as their phenotype is known [1, 8–13]. However, using bulk atlases for single-cell identity has mostly been overlooked. Instead, some studies have proposed to analyse bulk data using scRNA-seq data as a reference. For example, many deconvolution methods have been developed to estimate bulk sample cellular composition based on scRNA-seq [14, 15]. Only a few approaches have attempted to decipher cellular identity of scRNA-seq by leveraging bulk data. SingleR annotates query cells using labels of bulk reference samples that are matched to each cell according to Spearman correlation [16]. Capybara predicts continuous cell identity by regressing each query cell expression profile on a bulk reference with restricted linear regression [2]. SCRABBLE imputes scRNA-seq under the constraint that the averaged expression of imputed single cells is consistent with a given bulk reference [17]. To correct for batch effects in the query data, [18] projected scRNA-seq data onto a reference microarray dataset. These methods remain challenged by large technical differences between scRNA-seq and bulk data, in particular library size and zero composition [19]. [20] addressed this challenge by down sampling reads in reference bulk data prior to data integration with scRNA-seq query using the approach from Seurat [21].

**Figure 1:**
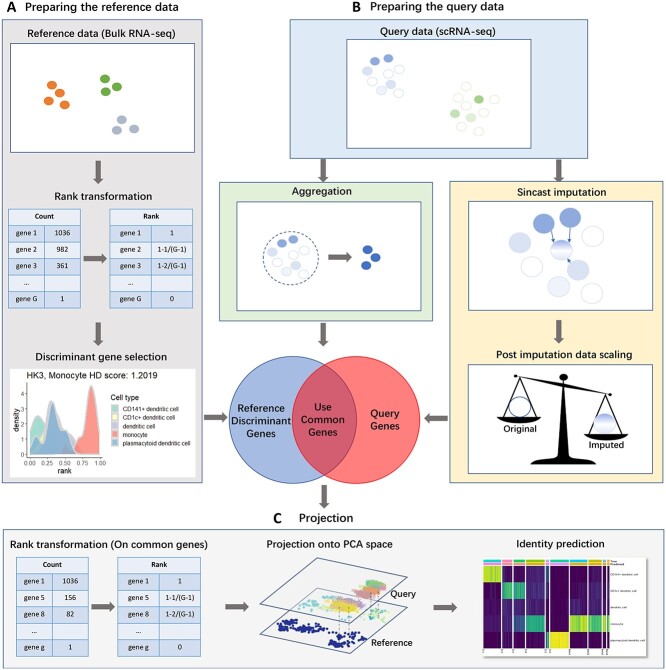
An overview of Sincast framework for projecting query scRNA-seq data onto reference bulk atlas. The differences in zero composition and scale between bulk and scRNA-seq data constitute major challenges to capture biologically relevant variation in the single cells, which Sincast addresses without data integration. (**A**) The reference bulk data are rank transformed, as proposed by (Angel et al.) 1 and additional gene filtering based on Hellinger Distance (HD) is applied to retain the most important genes discriminating cell types. (**B**) For the query single-cell data, Sincast either aggregates single cells by pooling the expression profiles of cells to create pseudo-bulk samples, or zero imputes the data by inferring unobserved expressions in a cell from the other cells in the query, followed by robust data normalization. The overlapping genes are then rank transformed for (**C**) projection, which consists in aligning both query and reference. PCA is performed on the reference data to construct a low dimensional expression space (atlas). Projection of the query is performed by calculating the query principal component scores learnt from the reference, and projection is further improved by diffusion mapDM. Cell identity prediction based on the neighboring reference samples on the atlas is performed with a modified Capybara score [2].

We propose Sincast (SINgle-cell data CASTing onto reference), a computational framework to query scRNA-seq data via projection onto bulk transcriptional reference atlases. Our framework avoids reliance on data integration to address technical differences across batches (Figure 1). Instead, we account for technical variation by normalizing data using rank transformation (RT) previously proposed in [1]. This transformation is highly scalable, applies independently to each sample and cell. Using RT, we can customize a comprehensive atlas by collecting and combing bulk samples from multiple sources, including both microarray and RNA-seq data. Atlases are built based on Principal Component Analysis (PCA). ScRNA-seq query data are projected onto the low-dimensional expression space spanned by the atlas principal components. The location of the query cells on the atlas allows the identification of similarities with well-annotated bulk reference samples. Prediction of cell identity is based on an improved Capybara score [2]. Most importantly, the core challenge of the structural differences between the reference and the query is addressed with two independent approaches, depending on the data structure of the query. We propose to either aggregate single cells to create pseudo-bulk samples, mimicking structure and variation of bulk samples, or to zero-impute single-cell data as sparsity is a major data characteristic that deviates single cell from bulk data. We rank transform the query and the atlas profiles independently, also avoid the need of batch effect correction. On five case studies (each query being projected on a relevant reference atlas), we demonstrate that we can robustly map single cells into correct biological niches of bulk atlases with a high concordance with the biology described in the original query study. The projection of imputed single cells also highlighted the value of bulk references in benchmarking single-cell computational methods.

## Results

### Projecting data after pseudo-bulk aggregation is a simple and effective way to reveal cell identity

Projecting single-cell data onto a bulk reference without addressing single-cell data sparsity performed poorly with Sincast. The projected cells tended to indistinctly cluster together toward the middle of the atlas relative to the locations of their biologically matching bulk samples (Supplementary Figure 1). This result was not surprising due to the large difference in data structure between single cell and bulk. In particular, a large number of zero values limits the linear separation of single cells on PCA.

Instead of direct bulk projection, we considered pseudo-bulk aggregation as a straightforward way to make single-cell data compatible for projection onto bulk. Aggregation is done by sampling cells of the same cluster with replacement and adding up their expression profiles. This approach is simple to implement and also conforms to our biological understanding that bulk expression represents pooled single-cell expression. We illustrate the usefulness of this approach through two case studies, where the query and reference data contain biologically matching cell types.

**Figure 2:**
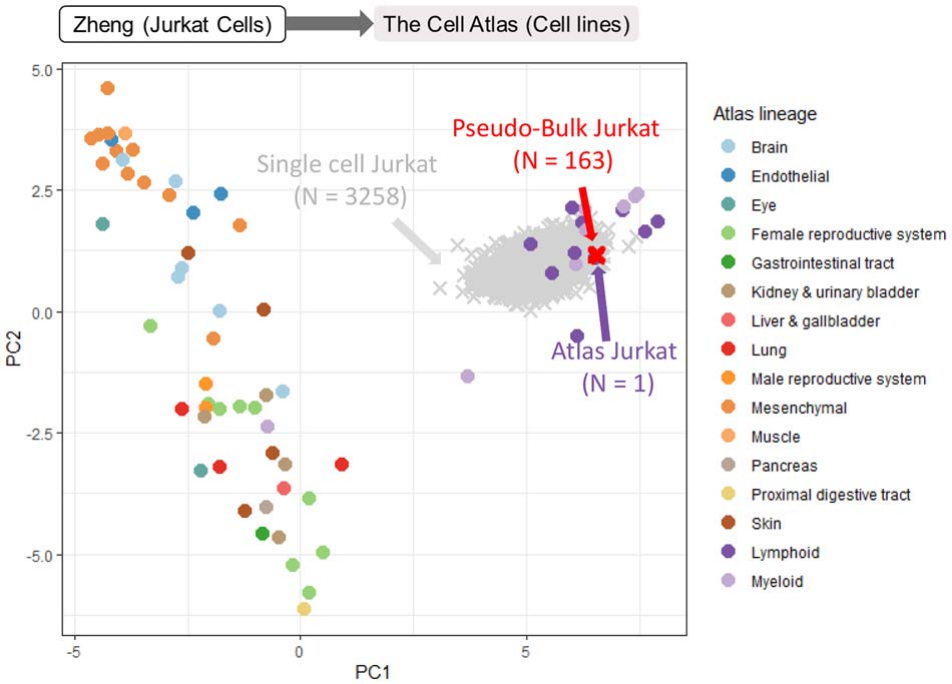
We projected single-cell data from [23], representing Jurkat cells profiled using 10x Genomics, onto The Cell Atlas [22] representing bulk RNA-Seq profiles of cell lines. Query cells were shown as crosses, and reference samples were shown as markers. Projection without any transformation resulted in the cells (in gray) being identified as lymphoid cells. After pseudo-bulk aggregation the cells (in red) projected closest to the Jurkat cells in the reference.

***Case study 1: Projecting Jurkat cells onto The Cell Atlas shows pseudo-bulk aggregation can classify cells accurately.*** The reference atlas from The Cell Atlas [22] consists of bulk RNA-seq data from a comprehensive range of cell lines. The query data from [23] contain Jurkat T cell line from 10x Genomics (32 058 cells, see also Table 1). PCA of the reference data showed a strong separation of blood cells from the other cell types along the first principal component (PC1, Figure 2). However, even though the nonaggregated single-cell data were projected onto the blood cell area of the PCA space, classifying them as one of the nearby cell types was difficult. Pseudo-bulk aggregation was more successful, as all aggregated cells were projected very closely to the Jurkat cell of the reference.

**Table 1:** Summary of the case studies, including the reference data on which we built the atlases and their number of samples, the query data for the corresponding reference atlases, and their numbers of cells, the number of discriminant genes selected for the reference atlas and their overlap between the reference and query prior to projection

**Reference data**	**Reference cells**	**Query Data**	**Query Cells**	**Genes Selected and Overlap**	**Used in**
[22] RNA-seq of 69 Cell lines	*N* = 69	[23] Single cell Jurkat T (10x v1)	N = 3,258	3,000/1,531	Section 2.1 Figure 2
[38] Gene expression data from the DICE project	*N* = 1,561	[23] single-cell Jurkat T (10x v1)	N = 3,258	2,000/1,556	Section S1.8 Figure S11
		[13] Bulk Jurkat T (Fantom5)	N = 1		
		[10] Bulk Jurkat T (ENCODE, identity: ENCSR000BXX)	N = 1		
[24] Molecular characterization of 29 immune cells within peripheral blood mononuclear cell.	*N* = 114	[25] Human immune response to COVID19 infection	N = 49,900	1,000/937	Section 2.1, S1.6 Figure 3 S2, S3, S1
[12] An integrated myeloid atlas	*N* = 901	[29] Deciphering human embryonic macrophage development	N = 1,231	2,000/1,952	Section 2.2 Figure 5 S4, S6, S12
Monocyte and DC subset of [12]	*N* = 500	[33] Human dedritic cell and monocyte subsets	N = 1,078	500/416	Section 2.2, S1.11 Figure 4 S5, S7, S13, S15
[37] Microdissected rat kidney tubules segments	*N* = 114	[36] Mouse Kidney cell scRNA-se	N = 5,000 sampled from 10,000 cells.	(a): 250/249. (b): 250/233	Section S1.9 Figure S16

**Figure 3:**
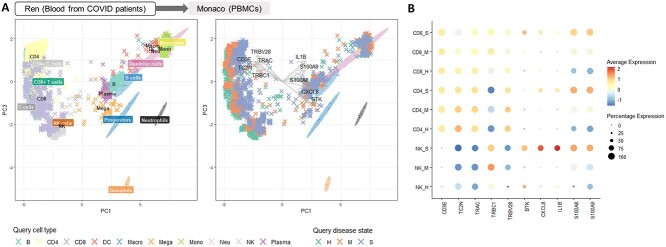
(**A**) We projected immune cells from COVID-19 infected donors as well as healthy controls [25] onto bulk RNA-seq atlas of immune cells [24] after pseudo-bulk aggregation. Query cells were shown as crosses. Reference samples were shown as ellipses and were labelled by colored boxes. PC3 is visualised as the first component that separates T cell subtypes on the y-axis. The cells were projected accurately onto the corresponding cell types of the reference (left). When we colored the same projected cells by disease state (right), we observed a clear shift in the identities of lymphoid cells according to disease severity (H: healthy, M: medium, S: severe). The arrows represent the top positive and negative loading of important genes that define PC1. (**B**) Dot plot showing the expression of the top loading genes as described in (B), highlighting an increase in the expression of each of the positive loading genes with disease severity.

**Figure 4:**
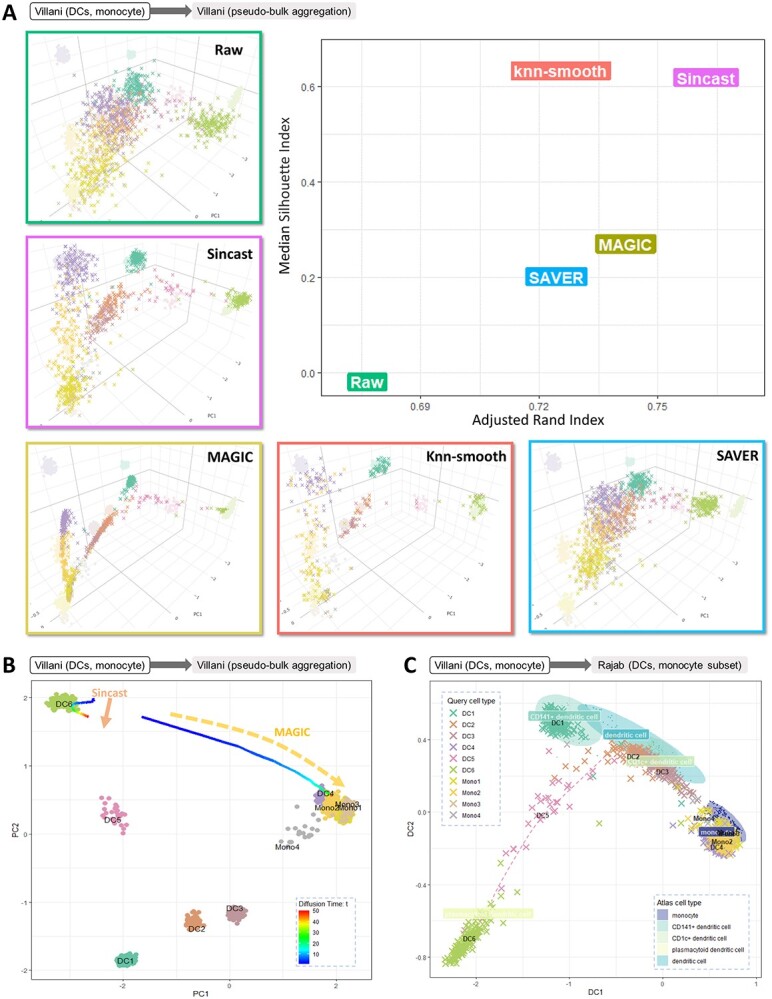
(**A**) We projected the DC cells from [33] onto a pseudo-bulk version of the same data to evaluate the performance of popular imputation methods in the context of projection. Measures of accuracy such as adjusted rand index and median silhouette showed Sincast performed best. (**B**) To assess impact of imputation tuning parameters on the projection results, we imputed then projected the subset of DC6, Mono1 and Mono2 cells from [33] onto the Villani pseudo-bulk atlas while varying the diffusion time parameter $t$ for MAGIC and Sincast. The line shows the centroids of projected points according to $t$ values. The DC6 population after MAGIC imputation was wrongly assigned monocyte identity when $t$ increased, unlike Sincast imputation that preserved the DC6 identity. (**C**) By reconstructing the PCA projection landscape with diffusion map (DM), Sincast imputed version of [33] projected the cells accurately onto the bulk DC and monocyte subset of [12]. The projection also highlighted the newly discovered DC5 population as a continuum state between pDCs and cDCs.

**Figure 5:**
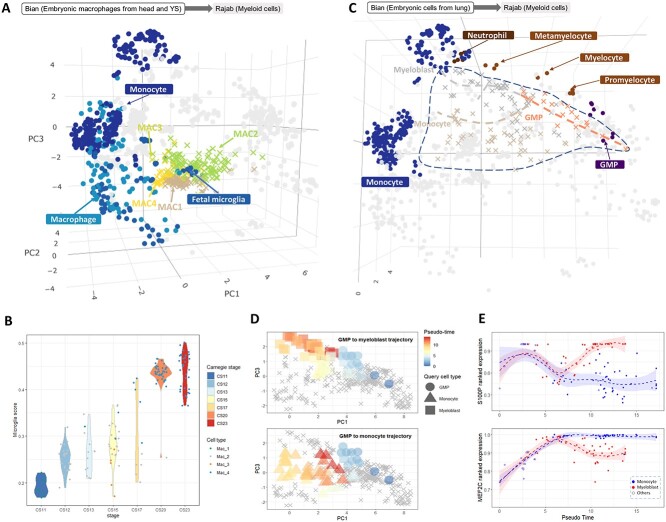
(**A**) Projecting embryonic macrophages from [29] onto [12] after Sincast imputation revealed their identity to be closest to the fetal microglia in the reference. (**B**) Sincast preserved the development trajectory inherent in the query data. Modified Capybara score of these cells against the reference microglia showed increasing values with their Carnegie stages. (**C**) Sincast workflow can produce pseudo-time trajectories. We applied a trajectory inference algorithm (Slingshot) to the projection of another subset of query data from [29] after Sincast imputation. This showed pseudo-time trajectories from GMPs toward either monocyte or myeloblast fates. (**D**) Projected cells colored by pseudo-time calculated from (C) showed a clear concordance with the annotated cell types in the query data. PC3 is the first component that shows the trajectory branches on the y-axis. (**E**) These trajectories can then be used to find key genes of differentiation. The expressions of neutrophil specific gene S100P (top) and monocyte specific gene MEF2C (bottom) were plotted against the pseudo-time values of the projected cells. These showed clear branching of their expression according to the cell fate.

***Case study 2: Querying COVID-19 case-control study data onto an immune cell atlas shows pseudo-bulk aggregation can highlight shifts in cell identity.*** The reference data from [24] consist of 29 immune cells sorted from peripheral blood mononuclear cells. The query cells were from [25], describing immune cells profiled on both healthy and COVID-19 infected donors. We selected nine donors from the same batch, in different disease stages of healthy, moderate and severe, to aggregate and project (see also Table 1).

Figure 3A illustrated the pseudo-bulk aggregated projection colored by cell type only (see Supplementary Figure 2 for the projection colored by atlas and query cell type). We observed a high concordance between query and reference cell types. Next, we colored the projected cells according to disease stage on the same plot. This projection illustrated that the T and the NK cell populations of COVID patients had identity shifts toward the positive direction of PC1 of the reference compared to the healthy controls (Figure 3A). We found that inflammatory markers such as BTK, CXCL8, IL1B, S100A8/9 were among the top 20 genes with the highest PC1 loadings (i.e. important genes that drive linear separation of samples on PC1). The shifts of cell population indicated an upregulation of these inflammatory signatures in COVID patients according to disease severity (Figure 3B). This finding was consistent with [25], who claimed that hyper-inflammatory cell subtypes defined by the systematical upregulation of these inflammatory signatures were one of the major causes of cytokine storm in severe COVID patients.

In the myeloid compartment of the projection, the shift in the projected monocytes of COVID patients compared with the healthy controls was difficult to visualize. Thus we applied our improved Capybara cell score to the projected cells [2] to quantify the projection more rigorously. Our predicted score revealed that non-classical monocytes (CD14- CD16+) in COVID patients acquired an intermediate monocyte (CD14+ CD16+) identity (Supplementary Figure 3), providing potential explanation on the reported increase of intermediate monocytes in the peripheral blood mononuclear cells (PBMCs) of COVID patients [26, 27].

This case study showed that pseudo-bulk aggregation can work beyond simply benchmarking cells when there is high concordance between the query and reference cell types and can reveal more intermediate cell types. It also illustrated how a projection method can rapidly generate biological insight, without the need to perform differential expression analysis separately for example. Indeed, the reference atlas already contained key genes that defined the principal components in the PCA space. Batch correction was not necessary when projecting, a feature from Sincast that provides a large advantage when the query data contain large batch effects. It is possible to extend this idea even further by using the reference as a background on which multiple query data can be compared with each other without batch correction (see Supplementary Material 1.8, Figure 11).

***Limitations of pseudo-bulk aggregation.*** Case study 2 (Figure 3A) highlighted some ‘mismatched’ cell projections near the centre of the PCA space, illustrating an inherent limitation of pseudo-bulk aggregation when the query cluster is highly sparse. Aggregation requires a sufficient number of bootstrap sampling from each cluster to overcome zero-inflation problem. Thus, a cluster composed of only a few cells poses a problem as the pooled gene counts may still be zero-inflated.

We defined sparsity in this context as the percentage of zeros present in a pseudo-bulk aggregated cluster. We assessed whether a sparsity threshold could indicate the appropriateness of pseudo-bulk aggregation, depending on the study and cell types. We down sampled the atlas samples to simulate sparse samples to project. The threshold was defined at the point where matched cell identities of sparse samples diverged (Supplemental Material 1.10). For example, case study 2 showed that any cluster with sparsity greater than 15 percent led to poor projection (Supplemental Figure 1, and Supplemental Figures 8, 9, 10, for other case studies).

### Data imputation prior to projection reveals complex single-cell biology

When the query data contain clusters with high sparsity or represent a more continuum of cell states rather than distinct states, data imputation offers an alternative to pseudo-bulk aggregation. However, we show that existing imputation methods created inaccurate projections, due to over smoothing of the query data prior to projection, resulting in over-shrinking the variance. Our imputation method builds on MAGIC [28] to project single-cell data onto bulk reference. We compare our method against existing imputation methods in two case studies and illustrate how imputation followed by projection can reveal new cell states.

***Case study 3: Existing scRNA-seq imputation methods show limitations when used to project onto bulk reference data.*** We considered the reference data from [12], where we previously integrated 44 microarray and bulk RNA-seq datasets to create an atlas of myeloid cells. The query data from [29] contain myeloid cells derived from human embryos (see also Table 1). Three existing single-cell imputation methods were compared with their default parameters: MAGIC [28], knn-smothing [30] and SAVER [31]. These methods chosen as they were the top three performers in the review of imputaton methods by [32].

We found that the projection of imputed single-cell data onto the reference differed greatly depending on the imputation method, reflecting the assumptions and characteristics of each method (Supplementary Figure 6). In this case study, cells imputed by MAGIC were connected to form smooth cellular trajectories with restricted local variance. Cells imputed by knn-smoothing were more scattered than MAGIC, as a result of iterative data aggregation during imputation. Cells imputed by SAVER, a model-based method that predicts the expression profile of each cell by regressing on the rest of the cells, were not shrunk locally relative to the global scale of the query data. The projection visualization can be used as preliminary benchmark to assess the relevance of these methods in this context.

To illustrate how these differences translated to specific projection results, we focused on the embryonic macrophages Mac_1 and Mac_4 in the query data. [29] noted that these describe distinct cell identities, where Mac_1 cells were mainly found in the yolk sac at Carnegie Stage 11, whereas Mac_4 cells were predominantly located in the head representing developing microglia. Only the projection made after MAGIC or Sincast imputation showed these cell types as distinct clusters (Figure 12).

***Case study 4: Sincast imputation produces more accurate projections onto bulk reference data.*** We next evaluated the performance of Sincast imputation against these three imputation methods. This case study used the query data from [33], which contain six dendritic cell (DC) subpopulations, fluorescence-activated cell (FACS) sorted and profiled using Smart-seq2 [34]. For the bulk reference, we chose a pseudo-bulk aggregated version of the query data itself and used the accompanying annotation as ground truth in the evaluation (see also Table 1). We also calculated median silhouette index (MSI) and adjusted rand index (ARI) on the query projection to evaluate the accuracy of the results. MSI and ARI measure how well each cell’s cluster membership is preserved before and after imputation.

While all imputation methods improved cell type classification compared to raw data projection. The failure of raw data projection suggests that single-cell data and bulk data are not directly comparable. Sincast imputed data performed best in terms of ARI and the second best in terms of MSI (Figure 4A). Each of the clusters of [33] projected onto their matched reference cell types after Sincast imputation. We then evaluated the robustness of Sincast regarding its imputation tuning parameters on the same atlas, compared with MAGIC (see Method Section 4.5). We only imputed and projected ten DC6 and 285 Mono1/Mono2 cells of the query (see Supplementary Material 1.11). We intentionally imputed each cell based on its 15 nearest neighbors (i.e a value larger than the actual DC6 population), and varied the diffusion time parameter $t$ for both MAGIC and Sincast before projection. With MAGIC, higher values of $t$ resulted in the DC6 cluster from the query data projecting further from the reference DC6 cell cluster, toward monocytes. We did not observe such effect with Sincast (Figure 4B).

Next, we queried Sincast imputed [33] data with the reference of the DC and monocyte subset from [12] (Supplementary Figure 7). We nonlinearly reconstructed the PCA projection landscape with DM, embedding the atlas samples and query cells into new data coordinates of diffusion components (Section 4.7). We found that DC5 cluster projected between conventional DCs and plasmacytoid DCs, suggesting a dual identity (Figure 4C, Supplementary Figure 5). This results was consistent with [33] who claimed that DC5 represent a new subpopulation of DCs, which lie on the continuum between these two states. This highlights how Sincast imputation and projection can reveal new cell states, which may not exist on the reference data.

***Sincast imputation can highlight pseudo-time trajectories.*** We considered a subset of data from [29] corresponding to macrophages from the embryonic head and york sac. The cells were projected onto [12] atlas after Sincast imputation. As expected, the cells were projected close to fetal microglia in the reference (Figure 5A). When we investigated our modified Capybara score for each of the projected cells against the reference microglia cell types, there was an increase of this score according to the Carnegie stage of the embryo (Figure 5B). This result showed that Sincast imputation followed by projection can preserve the inherent time course information in the query data.

We then considered a different subset of cells from the same query data, involved in the monocyte to neutrophil differentiation process in the lung, and projected these cells onto the same reference atlas after Sincast imputation. We ran the unsupervised trajectory inference algorithm Slingshot from (Street et al.) 35 on the PCA of the projected cells (Figure 5C). This analysis highlighted pseudo-time trajectories originating from granulocyte–monocyte progenitors (GMP) and branching toward the myeloblast and the monocyte cell fates (Figure 5D). When we identified the significant genes with loadings that are in the same directions of the trajectory development, they represented the typical marker genes that are associated terminal cell types of each trajectory (i.e. S100P for neutrophil, MEF2C for monocyte) (Figure 5E).

These examples showed that pesudo-time trajectories can be inferred correctly from the Sincast workflow. They also illustrate another major advantage in performing pseudo-time analysis after projecting onto a reference: only subsets of the query data are required, as the reference data already provide sufficient underlying structure for a trajectory analysis. In addition, without formal differential expression testing, key genes along a trajectory can be simply inferred by the gene loadings of principal components.

## Discussion

The analysis of scRNA-seq data requires unbiased characterization of the transcriptional identity of each cell. Even though many bulk RNA atlases have been developed over the decades—covering most tissue types and offering rich phenotype data such as FACS markers and extensive sample annotations, they have been currently ignored in cell type annotation and cell identity prediction tools. Our computational framework is designed specifically to leverage these well curated and established bulk transcriptional data as references. Sincast projects query scRNA-seq data onto the low-dimensional expression space learnt on the bulk reference using PCA. PCA preserves euclidean distances between cells and produces new data coordinates that are easy to interpret, compared with nonlinear data embedding methods such as UMAP, and is more suited to bulk data. When projected to the bulk atlas, the transcriptional identity of each single cell can be interpreted visually, based on its location on the atlas, but also quantitatively, using our improved Capybara cell score. Both approaches can reveal novel single-cell biology that can be defined as a composition of bulk biology, such as intermediate cell types, cell states and rare cell populations. For example, with PCA, transitioning cells can be identified when projected between major atlas cell clusters. With Capybara, transitioning cells will be assigned a high score on multiple atlas cell types corresponding to the root, branches and ends of the transition. Two query data processing pipelines are proposed, aggregation and imputation, to mitigate the structural discrepancy between bulk and scRNA-seq data in the projection result.

Our first approach, cell aggregation, generates *in silico* mimics of bulk RNA-seq samples and is primarily designed for recovering pseudo-bulk identities of cell populations in the query scRNA-seq data. Cell aggregation is easy to implement and preserves global scale and genuine population differences of the query data. Moreover, pseudo-bulk samples have valid statistical interpretation as they are built based on bootstrap sampling of query cluster averages. By visualising the degree of overlap between clusters of pseudo-bulk samples on the atlas, one can obtain a first understanding on whether clusters of cells differ significantly based on their averaged expression. Pseudo-bulk analysis is particularly suitable for case–control studies in which cluster level differences are of greater interest than of cellular level variation within clusters, as we showed in Case study 2. An additional use case for pseudo-bulk aggregation is the creation of a reference for evaluation of single-cell methods, as we showcased in Case study 4 with the [33] query for self-projection to evaluate imputation methods. Other use of pseudo-bulk aggregated data include appending an existing bulk atlas to extend its range of cell states. Sincast facilitates this process through its aggregation workflow.

However, aggregation also has its limitations as pooling and averaging ignores within cell cluster variation. As a consequence, meaningful sub-population signal detected by scRNA-seq can be masked in pseudo-bulk samples. For example, our attempt to project the [29] data was challenged due to the complexity of the study underlying biology (not shown). Continuous time resolution in cell development was lost, and the number of cells with a common combination of biological attributes (cell type, tissue location, development stage) was too small to generate valid pseudo-bulk samples. In that case, it is better to choose our cell imputation approach.

We compared the performance of Sincast imputation with three other popular scRNA-seq imputation methods: MAGIC, knn-smoothing and SAVER. We imputed the same query data with the methods’ default parameters. The query projections onto the bulk atlases resulted in different data structures and scales depending on how each method models cell-to-cell relationships. This comparison raised the issue that imputation may induce excessive technical artifacts. Thus, choosing a suitable imputation method with appropriate tuning parameters is important and should be evaluated with the overall aim of the analysis. Sincast imputation is designed to perform well with poor tuning or default parameters, and hence is accessible for users who are not familiar with the algorithm. However, the risk of over-imputation still exists. Other been used for projecting single-cell data onto bulk reference, Sincast imputation can also be extended for other types of analyses, such as clustering, differential expression analysis.

Regarding general guidelines for choosing between the imputation and the aggregation approaches, we propose the following. Best practice is to try both approaches as the resulting projection results can inform on the suitability of the approach. The aggregation approach applies when existing clustering assignment of the data are reliable and the aim is to benchmark overall cluster identity. Otherwise, cell imputation, which can model and retain complex cell-to-cell relationships in the scRNA-seq data, can be a better choice but can be computationally costly as memory usage grows in the order of $O(N^2)$, where $N$ is the number of cell in the data. For example, Sincast imputation on the [33] data (1078 cells and 416 genes) and [29] data (1231 cells and 1952 genes) with a laptop with 12 cores and 8.00 GB RAM took 1.37 seconds and 3.92 seconds respectively, and used 30.9MB and 54.6MB memory, respectively.

Query identity profiling was performed using an improved version of the Capybara cell score from [2], based on restricted linear regression. We chose Capybara for its ability in providing smooth quantitative profiling of single cells whose identities might be between the major cell types and states of the reference. Rank formation is also a perfect fit for a regression based method because ranking profiles are positive, with constant variance and scales. Other tools were considered, such as Machine Learning classifiers, but they tends to assign cells to specific (discrete) reference categories. However, since collinearity between the reference gene expression profiles affects linear regression models, all predicted cell type scores other than the dominant cell type should be considered when characterizing a query cell identity with our prediction tool. 

Finally, all our case studies were based on atlases of blood and immune cells, which are naturally separated in fluid tissues. In contrast, cells in solid tissues have been difficult to isolate in the past, thus reducing the ability to build quality reference atlases. One way to address this limitation is to aggregate well annotated single-cell data to build pseudo-bulk atlases. Aggregated cells have higher gene detection rates and hence larger statistical power for benchmarking query data. This approach in Sincast would also avoid integrating the reference and the query data. One example of the broad applicability of Sincast in querying cells of solid tissue and across species is given in Supplementary Material 1.9. We queried mouse kidney scRNA-seq data from [36] on the atlas built on micro-dissected rat kidney tubules segments from [37]. Our analysis shows that Sincast is able to handle related species while highlighting slightly different biology. We also identified potential promising genes associated cell type transition in kidney cell types.

In conclusion, leveraging established bulk transcriptional atlases as reference data for determining cell identity in scRNA-seq data can lead to powerful biological insights. Sincast is an unique toolkit specifically designed for this purpose, and can be used to comprehensively annotate matching cell states as well as discovering new states. Sincast also provides a novel framework for single-cell computational method evaluation. 

## Methods

### Data description

All data were collected from public data repositories, as described in Table 1.

### Building a bulk transcriptional reference atlas

We define bulk transcriptional reference atlas as a PCA representation of a gene expression dataset to which external data (i.e. scRNA-seq data) can be projected and queried. This section details the data pre-processing steps required to build the reference atlas prior to PCA (Figure 1), where we assume that quality controls on the reference data, such as low-quality gene and sample filtering have been performed.

We first perform RT to normalize the reference data, as previously described by [1], and further detailed in Supplementary Material 1.1). Only discriminant genes relevant for classifying the reference sample cell types (or any other class of interest) are selected to build the reference atlas (we summarize the number of genes retained in our case studies in Table 1). For data without distinct class assignment, one can either perform sample clustering on the data first, or use highly variable genes as substitute of discriminant genes [39]. We assess the relevance of a gene by calculating the correlation between the samples ranked expression of the gene and the samples (known) cell type labels, using the HD. Details on how to calculate the HD score can be found in Supplemental Material 1.2.

Sincast projection requires that the query genes match the set of genes used to construct the PCA reference atlas. Hence, overlapping discriminant genes are retained between reference and query. The reference data are rank then transformed again to adjust for the change of gene sets and the reduction of available ranking allocation. PCA with gene centering is then applied to the reference data to project samples into low-dimensional coordinates that maximize sample variation (as detailed in Supplementary Material 1.3).

### Projecting the query data onto the bulk reference atlas

We define projection as mapping query cells onto the PCA space of the reference atlas. This allows us to benchmark query biology by measuring the cell locations relative to the distributions of the reference samples from the atlas. RT followed by gene centering is applied to the filtered query data, where centering factors of the query genes are the same as from those of the reference data. We project the query cells by multiplying their centered rank profiles with gene loading matrix of the reference data, which defines rotation of gene coordinates to obtain atlas PC basis. Reference samples and query cells can then be visualized jointly on the atlas coordinates, where distances between samples and cells indicate their transcriptional profiles similarity. However, projecting sparse scRNA-seq query data onto bulk atlases is challenging, as RT is not sufficient for sparse data normalization. The large proportion of tied gene expression and inflated zeros violates the RT assumption of constant gene rankings across batches and libraries. We describe below how Sincast addresses this issue via pseudo-bulk aggregation and imputation on the query single-cell data before projection.

### Sincast pseudo-bulk aggregation

Cell aggregation has been used in single-cell studies to use bulk statistical methods, such as differential expression testing [40, 41]. In Sincast, we recommend using an aggregation approach when the query scRNA-seq data satisfy the following requirements:

1. Cells can be distinctly separated according to clusters. Cellular variation within cell clusters is not of primary interest, and cells are considered as pseudo replicates.
2. The unit of the query data must be additive (e.g. raw UMI count, TPM or CPM transformed data).

For the latter requirement, note that aggregating log-transformed counts is equivalent to multiplying counts and then performing log transformation; thus, the resulting aggregated samples do not represent valid bulk identities of cell populations.

We consider query data clustered according to cell types or other combination of identity labels of interest. We denote the number of cells of cell type $t$ as $N_{t}$. Aggregation is simply performed by sampling cells of cell type $t$ with replacement $N_{t}$ times, and then calculating the average expressions across re-sampled cells on a gene-by-gene basis to create pseudo-bulk samples. The sampling bootstrap procedure is repeated $B_{t}$ times for each cell type $t$ independently, where $B_{t}$ is usually chosen to be at least $N_{t}$. Labels of pseudo-bulk samples are inherited from the labels of single-cell cluster from which the samples are generated. Bootstrap sampling is often used for inferring sampling distribution of a given statistic. Here, the idea is to infer the sampling distribution of averaged expression profiles of single-cell populations.

### Existing imputation methods for scRNA-seq

RT is limited by small library sizes of scRNA-seq, resulting in many tied expressions and zeros to adequately align query scRNA-seq to reference bulk-seq data. One solution to address the structural discrepancy between the query the reference is to impute and smooth values in the query. Here we describe three best-performing scRNA-seq imputation approaches (evaluated by [32]) that were benchmarked in our study. MAGIC [28] in particular prompted the methodological development of Sincast.

***MAGIC*** (Markov Affinity-based Graph Imputation of Cells)) [28] is based on the theory of DM. MAGIC first computes a cell-wise distance matrix for the query data, then converts the distance into a probabilistic similarity measure called ‘affinity’ using adaptive Gaussian kernels. The affinity matrix is row-stochastic normalized into Markov transition matrix, whose entry represents transition probabilities from the row to the column cells. The imputed expression profile of a cell is the weighted average profile of cells within the targeted cell’s neighborhood where the weights correspond to the transition probabilities of the Markov matrix.

**Figure 6:**
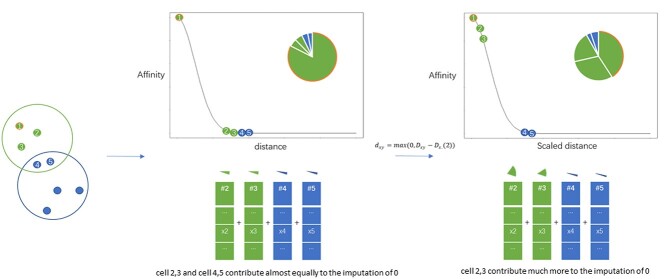
A schematic diagram showing MAGIC sensitivity to tuning parameters. Suppose the query contains two-cell populations represented as green and blue points and cell 1 is to be imputed. Using MAGIC affinity matrix specification, cell 4 and 5 contribute highly to the imputation of zeros in cell 1 if a wrong neighborhood size for imputation (5 in this case) is chosen. We propose to address this issue by scaling the distance measurement to highlight differences in distances, so that cells 2 and 3 participate more in the imputation.

The performance of MAGIC can be largely affected by the tuning parameters, primarily the exponent of the Markov matrix, called diffusion time $t$, the cell neighborhood size, knn-max and the bandwidth of diffusion kernel. The affinity between two cells that are not in each other’s knn-max neighborhood, is set to zero, which means that these two cells will not participate in each other imputation. When knn-max is set to a too small value, the imputed scRNA-seq data will retain a high proportion of zero expression value due to small pooling size. When knn-max is set to a too large value—larger than the cell population size, the cell is almost equally imputed by the other cells in its neighborhood, from the same or different types and states. This is a result of high dimensionality, where distances between cells to their neighbors are large and indistinguishable (Figure 6). This, in turn, make affinities among cells small and indistinguishable due to the fast decaying tail of the Gaussian kernel function. The impact of knn-max is further aggravated by increasing the imputation strength using the diffusion time $t$ parameter. Our proposed approach described next addresses these limitations.

***SAVER*** (Single-cell Analysis Via Expression Recovery) [31] assumes that the UMI counts of scRNA-seq data follow a negative binomial distribution framed as Poisson-Gamma mixture. SAVER performs penalized Poisson Lasso regression of each gene using the rest of the genes as predictors. The fitted regression values are set as prior Gamma means for the Poisson rate, and the Gamma variance is estimated empirically with a maximum likelihood approach. The final imputed value for each gene in each cell is the posterior mean of the Poisson rate, i.e. the weight between the regression fit and the empirical observation.

***knn-smoothing*** (K-nearest neighbor smoothing) [30] first aggregates the expression profile of each cell with its nearest neighbor to initialise the input cells for the next iteration. In the next iteration, the aggregated profiles are smoothed again, but this time each cell is aggregated with its three-nearest neighbors. The process iterates with increasing aggregation size equals to $2^i-1$ at $i^{th}$ iteration. The iteration stops when the aggregation size reaches a set maximum $k$.

### Imputation with Sincast: a graph-based approach

Our imputation method is inspired by MAGIC, and is modified on the theoretical basis of DM and UMAP—both are nonlinear data embedding methods that recover low-dimensional representation of the manifold underlying data in the euclidean space [28, 42, 43]. Our method aims to

1. Infer a $\kappa $-neighbor graph from the query scRNA-seq data based on UMAP (steps 1–4 in algorithm 0),
2. Construct a diffusion operator from the graph that is applied to the query for data diffusion (steps 5–8 in algorithm 0).

We assume that cells in the query can communicate and exchange their expression profile according to their local arrangement on the manifold. Gene expression of a cell is imputed as the weighted average gene expressions of the cell’s $\kappa $ nearest neighbors. Weight for imputation between a pair of cells is derived from their geodesic distance measured on the manifold. Our pseudo-code is presented in Algorithm 0.

***Distance scaling.*** Suppose $G$ (Gene) by $N$ (Cells) normalized gene expression matrix of the query data $X$. Consider $S = \{c_{1},c_{2},...,c_{N}\}$ as an ordered set that contains the column vectors of $X$. Cells $c_{i}$ in $S$ are assumed to be sampled from a low-dimensional manifold embedded within the data $\mathbb{R}^{G}$ expression space. We use a graph $\mathcal{G} = \{V, E, k\}$ to represent the pairwise geometric relationships of cells on the manifold. In such setting, cells can be considered as nodes of $\mathcal{G}$ ($V(\mathcal{G}) \ =\ S$), connected by weighted edges, whose weights $W_{ij}$ are given by the pre-defined kernel functions $k:\ S\times S\rightarrow R_{\geqslant 0}$, $k(c_{i},c_{j}) = k(c_{j},c_{i})$. The weight $W_{ij} = k(c_{i},c_{j})$ represents the similarity between cells $i$ and $j$ with respect to their geodesic distance on the manifold, where $k$ is derived from adaptive Gaussian kernels applied to pseudo-matrices defined individually for each cell $c_{i}$ in the query. Denote $knn(c_{i})=\{c_{i_{1}},c_{i_{2}},...,c_{i_{\kappa }}\}$ the set of $\kappa $ nearest neighbors of cell $c_{i}$. As we do not know the true structure of the underlying data manifold, the geodesic distance between $c_{i}$ and its $j^{th}$ nearest neighbours $c_{i_{j}} \in knn(c_{i})$ is approximated by the euclidean distance in $\mathbb{R}^{G}$ (valid only if $\kappa $ is small enough):

$$\begin{align*} &d_{\mathbb{R}^{G}}(c_{i},c_{i_{j}}) = \sqrt{\|c_{i}-c_{i_{j}}\|^{2}}. \end{align*}$$

The euclidean distance is then converted to cell-specific pseudo-metrices defined by the distance beyond nearest neighbor:

$$\begin{align*} &d_{c_{i}}(c_{i},c_{i_{j}}) = max(0, d_{\mathbb{R}^{G}}(c_{i},c_{i_{j}}) - d_{\mathbb{R}^{G}}(c_{i},c_{i_{2}})). \end{align*}$$

The reason for this step of distance scaling can be simplified as follows (for theoretical details, see [42])

1. Since now $d_{c_{i}}(c_{i},c_{i})$ and $d_{c_{i}}(c_{i},c_{i_{2}})$ are both 0 and indistinguishable, we can define a graph in which all cells are guaranteed to be locally connected to at least its first nearest neighbor. The weight of self-looping edge $(c_{i},c_{i})$ becomes less important compared with the weights of other edges $\{(c_{i},c_{j})| j\neq i\}$ connected to $c_{i}$. As such, neighbors of $c_{i}$ can contribute more to the inference of $c_{i}$’s identity, as we illustrated in Figure 6.
2. Because of the curse of dimensionality, distances between cells to their neighbours, based on their gene expression, are expected to show little variation relative to the absolute values of distances (i.e. $d_{\mathbb{R}^{G}}(c_{i},c_{i_{\kappa }}) \approx d_{\mathbb{R}^{G}}(c_{i},c_{i_{2}})$. We subtract the distance to each cell’s first nearest neighbor to mitigate that effect in the graph construction, and to put more emphasis on distances differences among neighbors.

***Weighted adjacency matrix.*** Next, we define the adaptive kernels $k_{c_{i}}(c_{i},c_{j})$ for $c_{i}$ as follows:

$$\begin{align*} &k_{c_{i}}( c_{i},c_{j}) \ =\ \begin{cases} exp\left( -\left(\frac{d_{ci}( c_{i},c_{j})}{\sigma _{c_{i}}}\right)^{2}\right) & c_{j} \in \ knn( c_{i})\\ 0 & c_{j} \notin \ knn( c_{i}) \end{cases} \end{align*}$$

The kernel bandwidth $\sigma _{c_{i}}$ is defined locally for $c_{i}$ with respect to the $c_{i}$ cell-specific pseudo-metric such that $k_{c_{i}}( c_{i},c_{i_{\kappa }}) = log(\frac{\kappa }{\kappa -1})$. The probabilistic interpretation for the choice of bandwidth is that all the cells in $X$ are set to communicate with their $\kappa ^{th}$ nearest neighbors with a fixed probability equal to $log(\frac{\kappa }{\kappa -1})$. Each cell’s bandwidth is derived from its distance to its $\kappa ^{th}$ nearest neighbor, which gives a proxy of the cell’s local density. Hence, by normalizing distances with local densities of cells, weights of connection between cells are defined irrespective of sampling density of the data.

We have already obtained a directed graph with asymmetric weighted adjacency matrix $W^{asy}$ whose entries are given by $W^{asy}_{ij} = k_{c_{i}}( c_{i},c_{j})$. However, asymmetric weights among different cells are not compatible as these weights are computed based on different matrices. To construct a valid Laplacian graph and hence a Markov transition matrix for data diffusion, we define a symmetric $W$ based on $W^{asy}$ to represent the final undirected graph $\mathcal{G}$:

$$\begin{align*} & W_{ij}= \frac{W_{ij}^{asy} + W_{ji}^{asy}}{2} * \frac{\sum _{k=1}^{N} W_{ik}^{asy} W_{jk}^{asy}}{\sum _{k=1}^{N} W_{ik}^{asy} +W_{jk}^{asy} -W_{ik}^{asy} W_{jk}^{asy}} \end{align*}$$

The term on right-hand side of the fraction product represents the Fuzzy Jaccard Index (FUJI, (Petković et al.) 44) measured between the knn graphs of cell $i$ and $j$. We modified FUJI by swapping the minimum t-norm on the numerator to a product t-norm and the maximum t-conorm at the denominator to a probabilistic t-conorm. Our graph is constructed to highlight the connection of cells that share common neighborhoods. The connectivity constrain down weights potentially poor connections in the graph and improve the robustness of the imputation.

***Data imputation.*** Using the theory of DM, $W$ is the diffusion matrix defined by $(S,k)$. Let $\overline{q}(c_{i}) = \sum ^{N}_{j=1} k( c_{i},c_{j}) = \sum ^{N}_{j=1} W_{ij}$ be the finite approximation of kernel volume (or degree in graph) for cell $i$. We define a new kernel scaled by the local volumes for Laplace–Beltrami diffusion,

$$\begin{align*} &\overline{W}_{ij}=\overline{k}( c_{i},c_{j}) = \frac{W_{ij}}{\overline{q}( c_{i})\overline{q}( c_{j})}, \end{align*}$$

and obtain the Markov transition matrix, or diffusion operator $P$ by row stochastic normalization:

$$\begin{align*} & P_{ij} =\frac{\overline{W}_{ij}}{\sum ^{N}_{j=1}\overline{W}_{ij}} \end{align*}$$

Data imputation is done by applying powered operator $P^{t}$ on $X$  

$$\begin{align*} & \overline{X} = XP^{t} \end{align*}$$

where $t$ is a positive scale parameter that controls the step size of diffusion random walk. A large $t$ value usually results in stronger imputation strength and less noisy data, and also over-imputation. The risk is a loss of biological signal as the Markov process may attract the identities of minor cell populations towards the regions in $\mathcal{G}$ with low escaping probabilities (these regions often correspond to discrete biological niches) in a long-time diffusion. 

***Visualization*** To get a sense of the geometry of the data that defines the graph used for data imputation, we can visualize the data embedding by mapping each cell $c_i$ to its first three diffusion coordinates

$$\begin{align*} & \Psi _{t}( c_{i}) \ =\ \left( \lambda ^{t}_{1} \psi _{1}( c_{i}),\ \lambda ^{t}_{2} \psi _{2}( c_{i}),\ \lambda ^{t}_{3} \psi _{3}( c_{i})\right),\end{align*}$$

where $\psi _1, \psi _2, \psi _3$ are the left eigenvectors of $P$ with the top three largest corresponding eigenvalues $1> \lambda _1 \geqslant \lambda _2 \geqslant \lambda _3 \geqslant 0$. These eigenvalues are only strictly less than 1 if the graph is connected. The constant eigenvector $\psi _0$ of $P$ with eigenvalue $\lambda _0 = 1$ is not of our interest and so is omitted from the visualization.

***Parameter tuning.*** By default, the graph of the query data is computed based the PCA of $X$ for dimension reduction and global noise filter prior to distance calculation (see Algorithm 0). By default, $\kappa = 30$. Two alternative ways of choosing $\kappa $ are also proposed based prior assumption on the characteristics of the data set:

*Option A.* We can approximate the minimum $\kappa $ that gives a connected graph. This approach is recommended when we assume that no cells or biological components in the data are functionally isolated.

*Option B.* We can approximate the minimum $\kappa $ to reduce the sparsity of the data to 25% when $t=1$. This approach avoids tuning $t$ in the imputation, but the euclidean distance may no longer be a valid approximation of geodesic distance when $\kappa $ is large. In A, if $\kappa $ is much larger than in B, the latter should be preferred.

We found that the parameter $t$ had a significant impact on the imputation result, based on our case studies: a large $t$ value tended to distort the data structure compared with an imputation with $t = 1$. For most of the query data we examined, a small $t\approx 3$ was usually enough to reconstruct complex cell-to-cell relationships with a wide range of $\kappa $ values. Regardless of the tuning of our parameters, we showed that our methodological improvements, such as using a distance beyond nearest neighbor and FUJI greatly compensated for a poor parameter choice, highlighting our algorithm’s robustness and accessibility for imputation and method evaluation.

***Data scaling after imputation.*** We found that nearest neighbor based graph imputation methods (e.g. MAGIC, knn-smoothing) can easily over-smooth the query data when the tuning parameters are not chosen carefully. For instance, the projection of query data imputed by MAGIC showed strongly reduced local variance and shrinking of the global structure relative to the atlas landscape when the diffusion time $t>1$. The loss of local variation is expected due to averaging gene expression of cells within each cell’s neighborhood. The shrinkage of query distribution towards its global average happens when the cells’ defined local neighbourhood sizes are larger then their actual size (as we showed when comparing the MAGIC and Sincast in Figure 4). To prevent overimputation and creating technical artifacts to the query data, we propose a scaling approach to shrink the imputed data back to the original data and recover part of the lost variance due to imputation. The degree of shrinkage in each cell is determined according to the amount of variation change in data due to imputation.

Briefly, we take the weighted average between each cell’s original and imputed expression profile as the data scaling result. Post-imputation data variance up-weights the imputed profile, whereas imputation strength measured by the deviation between the original and the imputed data up-weights the original profile (see Supplemental 1.4 for more details).


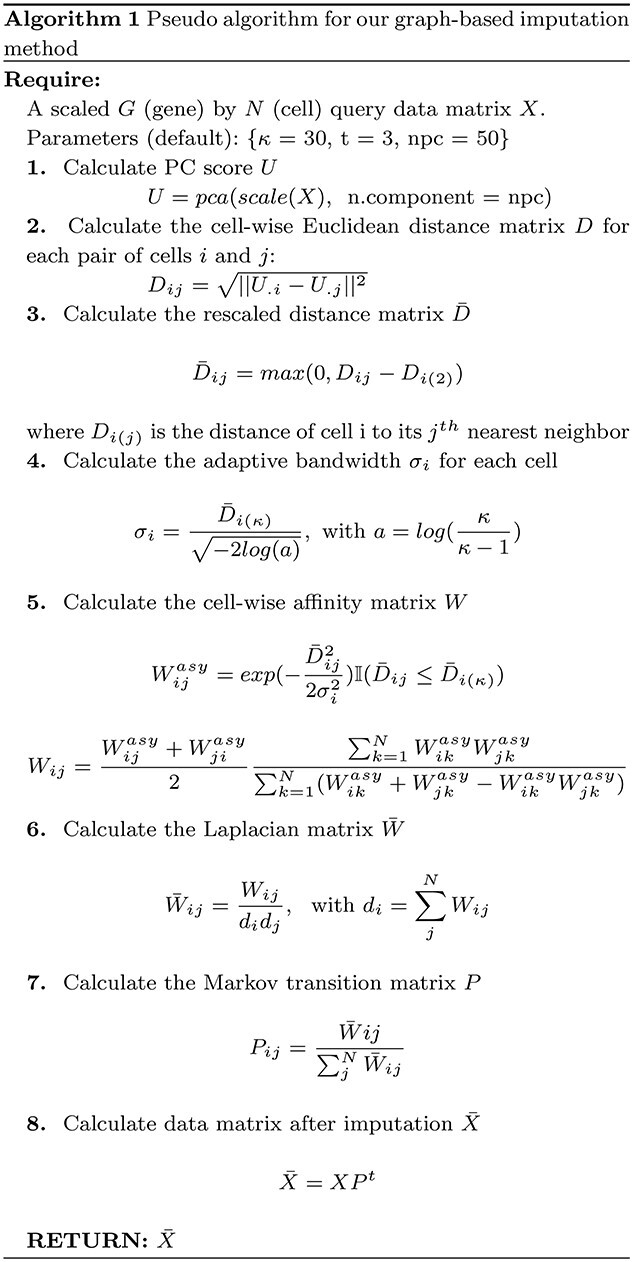


### Nonlinear visualization projection via DM

After projecting the query data onto reference atlases, we apply DM [43] to the concatenated PC scores (up to the elbow point) of query cells and reference samples to recover the manifold of the projection landscape. Indeed, we can only and practically visualize the first three PCs fitted on the reference samples, but these PCs only reveal the most important variations related to the reference biology, but not to the query. Query-specific but important information beyond the first three PCs can be missed. DM enables a fast, nonlinear reconstruction of the projection result, allowing for better visualization.

We used function *diffusion()* from the R package *diffusionMap* [45]. PHATE [46], a DM-based dimension reduction method can also be an alternative. Diffusion bandwidth in DM is data specific, set to be two times the maximum distance between the reference atlas sample pairs. We chose a large enough bandwidth to avoid creating a disconnected representation of the projection landscape. For a large integrated reference atlas rich in biological heterogeneity, a too small bandwidth only emphasises on the differences between atlas samples with distinct identities and will make the local views between single cells disproportionally smaller than the global view dominated by the atlas samples. As such, local views of projection will be difficult to visualize.

### Capybara cell score for continuum cell identity prediction

We applied the Capybara cell score (Capybara, (Kong et al. 2) to predict continuum identities of the query cells. Capybara performs restricted least square (RLS) regressions on each query cell transcriptional profile using cell type or cluster, averaged expressions of reference samples as predictors. Regression coefficients fitted for each predictor (cell type reference) correspond to identity score predictions. Capybara constraints the coefficient estimates on each query cell to be positive with total sum less than one for biological interpretation. We made two adjustments to improve the predictive performance of Capybara, as described below.

***Weighted RLS.*** Since different genes may have different degrees of contribution in explaining cell identities, we performed weighted RLS to assign observational weights to each gene corresponding to their importance in classifying cells. These weights (i.e. gene importance) can be estimated from the reference data in a various ways, including standardized gene variance, differential expression *P*-value or variable importance metrics from machine learning classifiers. We used gene HDs (also used for variable selection to build the atlas).

***Regression on neighboring samples.*** To take into account of biological heterogeneity in a comprehensive reference atlas, we propose to regress the query cell expression profiles on their neighboring samples within each atlas clusters, defined as the nearest sub-cluster medoids, rather than on the cluster averages (see Supplemental 1.5 for more details).

### Clustering assessment of query observations after projection

We used clustering performance of query projections on atlases as a mean to evaluate the goodness of projection. Clustering performances were quantified using the Silhouette Index, the Distance ratio and the ARI (see more details in Supplemental Material 1.7).

**Key Points**

- Sincast uses RT and discriminant gene filtering based on HD to build the reference bulk RNA-seq atlases.
- The query cells from scRNA-seq data can be either aggregated or zero imputed, without the need for batch effect correction.
- Single cells are projected on the reference bulk atlas using PCA and DM allows visualization across several PC dimensions.
- Cell prediction along a continuum allows to highlight new cell states.
- Key gene regulators can be identified as well as pseudo-time trajectories.

## Supplementary Material

Sincast_Suppl_bbac088

## Data Availability

Sincast R functions, code and data analyzed in this manuscript are available in https://github.com/meiosis97/Sincast.

## Author Biographies

**Yidi Deng** is a PhD candidate at the School of Mathematics and Statistics, the University of Melbourne, whose research interests are in developing statistical methods for single cell data integration analysis.

**Kim-Anh Lê Cao** is Associate Professor at the School of Mathematics and Statistics, University of Melbourne. Her laboratory focuses on the development of computational and multivariate data integration methods for feature selection applied to biological data.

**Jarny Choi** is a postdoctoral fellow at the Centre for Stem Cell Systems, the University of Melbourne. He works on transcriptional cellular identity and differentiation, especially within the field of haematopoieisis and stem cells.
