# Supplementary material for: Sincast: a computational framework to predict cell identities in single-cell transcriptomes using bulk atlases as references
Source: Brief Bioinform. 2022 Mar 31;23(3):bbac088. doi: 10.1093/bib/bbac088 (PMC9155616; doi:10.1093/bib/bbac088)
Supplement: Sincast_Suppl_bbac088 [file sincast_suppl_bbac088.pdf]

1 Supplemental Text

1.1 Sample-wise data normalization with rank transformation

We used rank statistics to address technical variation in transcriptomic data analysis, similar to the approaches of Bolstad et al. [1], Angel et al. [2], Tang et al. [3]. Here, absolute gene expression values are first transformed to within sample rank percentiles. Previously, we showed that such Rank Transformation (RT) is a simple but robust data normalization technique which can correct for library size differences and account for the presence of technical variation due to sequencing platforms (batch effects) in a dataset that combines independent studies [2]. RT is applied independently on each sample expression profile. This provides flexibility in appending any extra sample to the reference without re-processing the entire reference dataset. Moreover, RT fits the data of different size and scales, including pre-normalized data publicly available, enabling to customize suitable reference atlases.

RT assumes that technical variation barely changes the relative expression levels of genes within samples (see Angel et al. [2]). We will briefly describe how RT is performed in Sincast. We first rank the absolute expression values of genes within each sample. The gene with the highest expression level in a sample is assigned to a value equals to the total number of genes in the data, denoted G. The gene with the lowest expression level is assigned 1. Ties in expression were equally ranked as if they were the lowest member of the tie. All rank values R_{ij} for gene i and sample j are then scaled to $(R_{ij} - 1)/(G - 1)$ so that gene expression values are distributed across $[0, 1]$ in each sample.

1.2 HD score to identify discriminant genes

We first discretize the gene ranks into T categories, where T denotes the number of unique cell types and is defined using gene-wise k-mean clustering. This facilitates comparisons between gene expression and cell type distributions using metrics developed for categorical attributes. One such metric is the Hellinger distance (HD), a measure of divergence between two probability distributions [4, 5]. Cieslak et al. [5] proposed to use HD in binary decision tree to determine the optimal tree split. A good split can create child nodes on which two class labels separate distinctly with little affinity shares between distributions whereby nodes are considered as the support. HD is calculated between the distributions of class labels: the higher the HD, the purer the nodes, and the better the split. In Sincast we use HD to quantify the purity of sample cell types on the unsupervised partitions of genes (analogue to nodes splitting) to assess the genes' predictive ability.

We calculate the genes HD scores for each cell type using one (labelled class $+$) versus the rest (labelled class $-$) approach. Consequently, each gene is assigned T HD scores

that represent its ability in predicting T cell types. A gene's relevance in classifying cell types is defined its mean HD score. Formally, for each gene g HD score for cell type t is:

$$D_H\left(t^+, t^- | P^g\right) = \sqrt{\sum_{i=1}^{T}\left(\sqrt{\frac{|P_i^g \cap t^+|}{|P_i^g|}} - \sqrt{\frac{|P_i^g \cap t^-|}{|P_i^g|}}\right)^2}$$

where t^+ is the set of samples labelled with cell type t, and t^- denotes the rest of the samples, P_i^g is the ith partition of gene g. By default we select the top 2000 ranked genes with the highest mean HD scores as markers to build atlases. Mean HD scores are also used as gene weights for the improved Capybara cell identity prediction, as described in Section 4.8.

Other metrics such as Information Gain or Gini Index could have been chosen, but are sensitive to class size imbalance and may result in gene selection bias.

1.3 Building a bulk reference atlas with PCA

PCA with gene centering is applied on the processed reference data. PCA generates a loading matrix that specifies the rotation of gene coordinates to define the atlas spanned by the principal components (PC) basis. The PC basis represents the latent dimensions embedded in the gene expression space that can capture maximal variations of the samples. Locations of samples in those dimensions known as component scores were computed by multiplying the loading matrix with the data matrix. As a result of matrix multiplication, linear combination of genes realizes rotation and creates components. The number of latent components to include in the atlas is automatically determined by the elbow method, which consists of finding the points of maximum curvature (elbow point) of the changes in cumulative explained variance of PCA with increasing dimensions. The 'elbow point' indicates that adding extra components does not significantly increase the variance explained in the data. However, to prevent missing potential subtle biological variations, five components after the elbow point were also added in the atlas.

1.4 Post imputation data scaling

First, for a given gene g and cell i in the imputed data $\overline{X}$, we scale its expression value with gene-wise scaling factors f_g:

$$\overline{X}_{gi}^{gws} = f_g \overline{X}_{gi}$$

where

$$f_g = \frac{\underset{i:\overline{X}_{gi}>0}{median}(X_{gi})}{\underset{i:\overline{X}_{gi}>0}{median}(\overline{X}_{gi})}$$

The scaling factors are chosen so that for each gene, cells with observed expressions can roughly retain the same location (in the statistical sense) after imputation. This first

scaling step is only valid when both X and $\overline{X}$ are non-negative, and zeros in the data represent missing values of expression.

Second, we assume that the uncertainty (or variability) of $\overline{X}_{gi}^{gws}$ is regularized by the gene g underlying mean of imputed expressions. The form of the regularization is modelled as a global dispersion trend of $\overline{X}^{gws}$, inferred by fitting a generalized additive model (GAM) on the log-transformed gene-wise mean $\hat{m}_g$, and variance $\hat{v}_g$ estimations. We assume that $\overline{X}_{gi}^{gws}$ is generated from a gene-wise truncated normal distribution bounded below, where zeros in the imputed data are modeled as weak biological signals that cannot be captured by sequencers and hence censored from observation. The estimation of $\hat{m}_g$ and $\hat{v}_g$ is calculated based on quantile-quantile regression minimizing the following objective function:

$$(\hat{m}_g, \hat{v}_g) := argmin_{(m_g, v_g)} \sum_{q:\overline{X}_{gq}>0} (\overline{X}_{gq}^{gws} - m_g + \sqrt{v_g} z_q)^2,$$

where $\overline{X}_{gq}^{gws}$ and z_q are q^{th} quantile for $\overline{X}_{g.}^{gws}$ and standard normal Z respectively. We use the *mgcv* R package [6]. Our GAM model has the following specification:

$$log(\hat{v}_g) \sim \beta_g \ + \ s\big(log(\hat{m}_g)\big)$$

where s is the smooth function that defines the cubic regression splines. To prevent overfitting, the basis dimension of the regression spline is chosen as the smallest value which passes the test of *k.check()* function in *mgcv*. Regression weights $w_g = 1/\sum_{i=1}^{N} \mathbb{1}(X_{gi} > 0)$ are also considered in this model to account for the uncertainties in model estimation induced by the sparsity of the data. Genes with high sparsity will participate less in the estimation of the global dispersion trend (See Supplementary Figure 14 for trends estimated).

Finally, for $\overline{X}_{gi}^{gws} > 0$, the observational variance $\hat{\delta}_{gi}$ is then approximated by the fitted global dispersion trend as

$$\hat{\delta}_{gi} = exp\left\{\hat{\beta}_g + \hat{s}\big(log(\overline{X}_{gi}^{gws})\big)\right\}.$$

We define the squared imputation residual in each observation as

$$\hat{e}_{gi} = (X_{gi} - \overline{X}_{gi}^{gws})^2,$$

which represents the strength of imputation on the observation. The weighted average between X_{gi} and $\overline{X}_{gi}^{gws}$ is the final output, and is defined as

$$\overline{X}_{gi}^{wt} = \hat{\lambda}_i \overline{X}_{gi}^{gws} + (1 - \hat{\lambda}_i) X_{gi},$$

with

$$\hat{\lambda}_i = \underset{g:\overline{X}_{gi}^{gws}>0}{mean} \left(\frac{\hat{\delta}_{gi}}{\hat{\delta}_{gi} + \hat{e}_{gi}}\right)$$

The weight $\hat{\lambda}_i$ is the mean estimation of imputation's impact on gene variation in cell i, and can be interpreted as follows: When $\hat{e}_{gi} > \hat{\delta}_{gi}$, the weighting procedure favors the

original data due to a large imputation strength and small post-imputation data variance, suggesting over-smoothing. When $\hat{e}_{gi} < \hat{\delta}_{gi}$, which is typically the case for genes with high sparsity, scaling encourages the imputation of zeros. Supplementary Figure 15B shows that our Sincast data scaling also works on MAGIC imputed data. $\hat{\lambda}$ also provides a good indication regarding potential over-smoothing (Supplementary Figure 15C,D).

1.5 Capybara cell score

For each cell type cluster t of size n_t in the atlas, we first perform Partition Around Medoids clustering on the atlas PC basis to find 5 partitions of the cluster represented by the partition medoids. Each sub-cluster, therefore, contained $n_t/5$ cells in average. Given a query cell c projected onto the atlas, we then searched for its nearest medoid in t. Back to the high dimensional space, we denote the ranking expression of the nearest medoid found for c as M_t^c. A cell specific reference matrix $M^c = [M_1^c, M_2^c, ..., M_T^c]$ was built, where T represents the number of reference cell type to benchmark the query against. Let y^c be the ranking expression of c, our goal was to solve the following least square problem,

$$argmin_{\beta^c}\ (y^c - M^c\beta^c)'\, W\, (y^c - M^c\beta^c)$$

subject to

$$I_T\beta^c \geqslant 0$$

$$\mathbf{1}_T\beta^c = 1$$

where I_T is a identity matrix of size T, $\mathbf{1}_T$ is a row vector of ones, and W is a diagonal weight matrix of size G, whose diagonal entries incorporate gene importance to the estimation of β^c. Denote the objective function above as $f(\beta^c)$, we observed that

$$f(\beta^c) = \beta^{c\prime}M^{c\prime}WM^c\beta^c - 2y^{c\prime}WM^c\beta^c + const$$

so that

$$argmin_{\beta^c}\ \frac{1}{2}\beta^{c\prime}M^{c\prime}WM^c\beta^c - y^{c\prime}WM^c\beta^c$$

subject to the same regression constrains is equivalent to the least square problem. The updated objective function is in the form of quadratic programming, and so can be solved explicitly. The solution $\hat{\beta}_t^c$ gave the Capybara cell score for cell c on cell type t.

1.6 Down sampling bulk samples to simulate sparse rank expression

We assume that when the total genetic material in a bulk sample reduces, the observed absolute expressions of genes in a sample gradually decreases to zero. Hence we generated the rank profile of a sample with a sparsity rate of p/G by substituting the ranks of the first p lowest expressed genes to 0, while keeping the rest of genes ranks unchanged. The sparse samples generated were then interpreted as pseudo-single cells. However, our simulation procedure cannot model variation loss in single cells due to rank ties that

were not zero expressions. To quantify such, we propose an *effective sparsity* metric, as we describe next in Section 1.7.

1.7 Metrics

Effective sparsity Let R_{ij} be the ith rank of ith gene in cell j. The effective sparsity of cell j is defined as

$$1 - \frac{\sum_{i=1}^{G} \mathbb{1}(R_{ij} \neq 1)}{G} \frac{\sum_{i=1}^{G}(R_{ij} - 1)}{\sum_{r=0}^{G-1} r\mathbb{1}(R_{ij} \neq 1)}$$

where the left of the fraction product calculates the proportion of gene expressed in cell i, adjusted by the factor on the right whose numerator calculates the observed sum of ranks in cell j, and the denominator gives the maximum possible sum of ranks on expressed genes when all of them are distinctly expressed.

Silhouette index *Silhouette index* (SI) measures how well cells were separated according to a known clustering assignment [7]. Clustering assignments of query cells were assigned the original cell type labels from the query study. For each projected cell i, we measured its average inter cluster distance

$$a(i) = \frac{1}{|C_i| - 1} \int_{j \in C_i, j \neq i} d(i, j)$$

where $|C_i|$ is the size of the cluster to which i belongs, and $d(i, j)$ is the Euclidean distance between cells i and j from the same cluster calculated on the atlas' PCs. We also measured the minimum average intra cluster distance of cell i as:

$$b(i) = \min_{k \neq i} \frac{1}{|C_k|} \int_{j \in C_k} d(i, j)$$

where C_k is a cluster which is distinct from C_i. The SI for cell i is then defined as

$$s(i) = \frac{b(i) - a(i)}{max(a(i), b(i))}.$$

SI ranges between [-1, 1] with larger value indicating that a cell is more similar to the other cells within the same cluster than the cells outside that cluster. In Section 2.2 for Case study 4, SI for each query cell was calculated with respect to the atlas samples because the query true atlas correspondence was known for the pseudo-bulk atlas. The i index represents the target query cell, $\{j | j \in C_i, j \neq i\}$ are the samples from the same cluster as i in the atlas and $\{C_k | k \neq i\}$ represent the remaining atlas clusters.

Distance ratio When assessing the impact of sparsity on query projection in Section 2.1, we defined the minimum inter cluster distance as

$$a_{min}(i) = min_{j \in C_i, j \neq i} d(i, j)$$

and the minimum intra cluster distance as

$$b_{min}(i) = min_{j \in C_k, k \neq i} d(i, j),$$

using the same notations as in the SI calculation. Distance ratios of pseudo-single cell i were then calculated by

$$r(i) = \frac{a_{min}(i)}{b_{min}(i)}.$$

We chose the minimum distance as the measure of cluster affinity to account for heterogeneous within-cluster variation in bulk atlases.

Adjusted Rand Index *Adjusted Rand Index*(ARI) was used to calculate the concordance between two sets of cluster assignments [8]. We performed cluster analysis on top PCs of the projected query cells and compare the result with the query's original cluster labels obtained by the query study. We chose k-mean clustering analysis, where k was set to be the same as the number of original query clusters. Rand index (RI) was then calculated between the two sets of cluster assignments, and was adjusted to correct for the possibility of obtaining the RI by chance. ARI ranges from 0 to 1 with a larger value suggesting that the k-mean clustering of projected observations resembles the query's original cluster assignments. Hence, we recruited ARI to quantify whether unsupervised clustering methods represented by k-mean clustering can still perform well on the projection of imputed query data. K-mean clustering was implemented using the 'eclust' function from the R package `factoextra` [9]. ARI was calculated using the 'cluster.stat' function from the R package `fpc` [10].

1.8 Querying Jurkat cell line on the Schmiedel atlas

To test Sincast when the query and the reference data are not compatible in biology, we build an atlas with the reference data from Schmiedel et al. [11], and we queried the pseudo-bulk sample aggregated by single cells of the Jurkat-T cell line from 10x Genomics [12]. We also used two additional bulk RNA-seq Jurkat samples (from FANTOM5 [13] and ENCODE projects [14], identity: ENCSR000BX) as query data acting as further benchmarks for the pseudo-bulk projection (see also Table 1).

Without batch correction, query Jurkat single cells, bulk and pseudo-bulk samples were projected to the same region at the middle of the Schmiedel et al. [11] atlas, being away from all the atlas clusters (Supplementary Figure 11A). Such query arrangement suggests that the query data shares limited transcriptional variation in common with the reference data. We used Capybara cell score to quantify the query identity (Supplementary Figure 11B). In average, the reference could only explain about 25 percent of variation in raw, unaggregated single cells. In contrast, the pseudo-bulk sample had about 60 percent of variation explained, same as which of the real bulk samples. In either cases of raw single cells, bulk and pseudo-bulk samples, the majority of the query variation was explained by the Activated naive CD8+ T cells (CD8-TA) of the reference. Enrichment analysis showed that CD8-TA was enriched in biological process and functions related to chemokine receptors, cytokine production, and T cell signaling (Supplementary Figure

11D). Characteristic genes of Jurkat T cells such as Interleukin 2 (IL2) were in the top list of differentially expressed genes of CD8-*TA* (Supplementary Figure 11C). Thus, CD8-*TA* is a good reference vocabulary representing Jurkat T cells. Consistency of profiling results on bulk and pseudo-bulk samples suggests that Sincast prediction is robust to query data's batch sources. When the query and the reference data are not consistent, Sincast can pick out the best reference vocabularies characterizing the query while quantifying the prediction uncertainty.

1.9 Querying mouse kidney single cell on microdissected rat kidney tubules segments

We evaluated Sincast performance to query biology from solid tissue. We built a reference bulk atlas with microdissected rat kidney tubules segments from [15], and queried mouse kidney single cell data from [16]. In this example, we used closely related but different species for the reference and query species and assessed the potential of Sincast for cross-species queries.

The genes in the reference data were pre-filtered in two different ways: **(a)** with 250 genes discriminating the atlas cell types according to their HD score (Supplementary Material 1.2). **(b)** 250 mouse kidney cell type markers provided by [16]. This set of markers is composed of the top 40 differentially expressed genes (DEG) of each tissue cell type (some cell type did not have 40 DEGs). The references from (a) and (b) were further filtered to retain only 249 and 233 homologous genes that were expressed in the query, respectively.

Since the reference data was slightly sparse, we then built the atlas by first aggregating the reference samples and then run PCA. Query data were imputed by Sincast with default tuning and projected onto each atlas of (a) and (b). In Supplementary Figure 16A), we observed that the projection in (a) underfitted the atlas as the query cells clustered towards the middle of the atlas relative to the atlas samples. The projection in (b) overfitted the atlas as the query cells exceeded the distribution range of the atlas samples (Supplementary Figure 16B). Underfitting in the atlas (a) suggests that the query cells cannot be separated according to the reference specific markers, while overfitting in atlas (b) suggests that the query cells include stronger signals on query-specific marker genes compared to the reference samples. Nonetheless, either case shows that the query cells are projected in the correct directions towards their biologically matching bulk samples. From this analysis, we can conclude that related species such as mouse and rat share slightly different kidney biology.

We next focused on the relevance of the genes highlighted from the projection on atlas (a). Park et al. [16] previously identified a transition from intercalated cells (IC) towards principal cells (PC) mediated by Notch signalling pathway. Inducing Notch gene expression shifts the transition towards the PC fate, which is potentially responsible for causing metabolic acidosis in mouse models and chronic kidney disease in human. Similarly, we also identified the IC to PC transition along the positive direction of Principal Components 1 and 3, meaning that genes of high loadings on these components might be associated with this observed transition. The loading plots in Supplementary

Figure 16C highlighted genes such as Nckap1, Krt18, Ptgs1 as potential candidates.

One of these genes, Ptgs1, Prostaglandin-endoperoxide synthase, produces prostaglandin, which had already been reported as a major metabolite of arachidonic acid in kidney, and a critical mediator of water and NA^{+} transport in connecting duct [17, 18]. Specifically, PC are responsible for water and electrolytes regulation. As expected, we observed an increase of expression of of Ptgs1 along the trajectory from IC to PC (Supplementary Figure 16D,E). We observed similar patterns of expression with Nckap1 and Krt18. Krt18 is a cell proliferation marker, which might also play a role in the IC to PC transition [19, 20]). In addition, Prostaglandin E synthase, Ptges, the terminal enzyme at the biosynthetic pathway of the most abundant Prostaglandin, Prostaglandin E_2, was also up-regulated during transition (Supplementary Figure 16D,E). Therefore, our Sincast projection analysis identified a potential association between Prostaglandin production and IC to PC transition.

1.10 Impact of sparsity on query projections

The maximum allowed sparsity rate (MAS) for scRNA-seq query projections is atlas and cell type specific. We demonstrate that the MAS for querying on an atlas can be approximated empirically by down-sampling the reference samples' expression profiles, simulating sparse samples that are then projected and the projections compared with their original locations on the atlas. Down-sampling was performed by gradually substituting ranks of genes in a sample to zero by the order of gene rank (Supplementary Material 1.6). We first analysed the Monaco et al. [21] atlas built for the Ren et al. [22] query task as an example (Section 2.1, Case study 2). Sparse samples were generated at sparsity rates from 0 to 100 percent with a step of 5. We treated sparse samples for each sparsity rate as pseudo-single cell queries with known atlas correspondence. Centroids of pseudo-single cell cell type clusters were depicted on the atlas in Supplementary Figure 1A. The trajectories of the centroids with various sparsity rate indicated identity shifts of down-sampled bulk samples. Separations of clusters were enhanced when sparsity rate was less than 40 percent. As sparsity rate increased, clusters tended to gather towards the centre of the atlas, indicating a loss of cell identity signal. We assessed the cluster trajectories quantitatively with each pseudo-single cell's minimum inter-cluster distance to minimum intra-cluster distance ratio, where the cluster of a pseudo-single cell is defined as its corresponding atlas cluster (Supplementary Material 1.7, Supplementary Figure 1B). Cluster trajectories were welly resembled by the distance ratio, which increase, decrease and then increase on the heatmap with increasing sparsity, describing how clusters moved away, back, and then away respectively. For the Monaco et al. [21] atlas, the suggested MAS where 90 percent of the sparse sample retain a distance ratio greater than 0.5 was 15 percent. For the other atlases, the MAS varied between 10 and 15 percent (See Supplementary Figure 8, 9 and 10).

Here we only assessed the impact of sparsity on single cell projection. Small gene counts are also enriched in single cells, and may technically affect the projection results due to their ties in gene ranking. The observed sparsity of a cell should therefore be adjusted by the cell additional variation loss in gene expression rank ties. We proposed effective sparsity to account for such variation loss (Supplemmentary material

1.7, Effective sparsity). As an example, the raw single cell data of Ren et al. [22] were projected accurately on the simulated cluster trajectory of the Monaco et al. [21] atlas (Supplementary Figure 1A). Effective sparsities of the query cells matched the sparsities of the simulated clusters to which the cells were projected, suggesting that the deviation of the query projection from the atlas clusters was driven by sparsity, not biology.

1.11 Sincast and MAGIC sensitivity to parameter tuning

As described in Supplementary Material 4.5, knn-max has a large impact on MAGIC imputation performance. We illustrated this issue with Villani et al. [23] data by subsampling the data to 10 DC6 (phenotypically pDC) and 285 Mono1/Mono2 subset (phenotypically classical/non-classical monocytes) (Figure 4.A). We fixed knn-max to 15, and performed MAGIC imputation with a grid of t increasing from 1 to 50 with a step of 1. DC6 imputed at each t were projected onto the [24] Mono-DC sub-atlas, and the centroid of each projection were shown. The result demonstrates that the identity of DC6 population was clearly distorted after MAGIC imputation. As t increasds, the DC6 cluster centroid was projected away from the atlas pDC niches towards the monocyte niches. However, after increasing the DC6 population to 20, the imputed DC6 cells were projected closer to the atlas pDC niches, suggesting that the DC6 population identity was recovered (Figure 13).

To mitigate the impact of poor tuning on imputation, we modified the knn-graph construction in MAGIC based on the theory of UMAP (Figure 4). We also proposed post-imputation data scaling to shrink the imputation result back to the original observations and prevent over-smoothing (see section 4.6 for a detailed description). We performed similar analyses as described above for Sincast method with local neighborhood size set to 15 ($\kappa = 15$, equivalent to knn-max = 15 in MAGIC). We observed that the shifts of the DC6 clusters towards the atlas monocyte population were greatly restrained even when the parameter tuning was mispecified (Figure 4.A).

2 Supplemental Figures

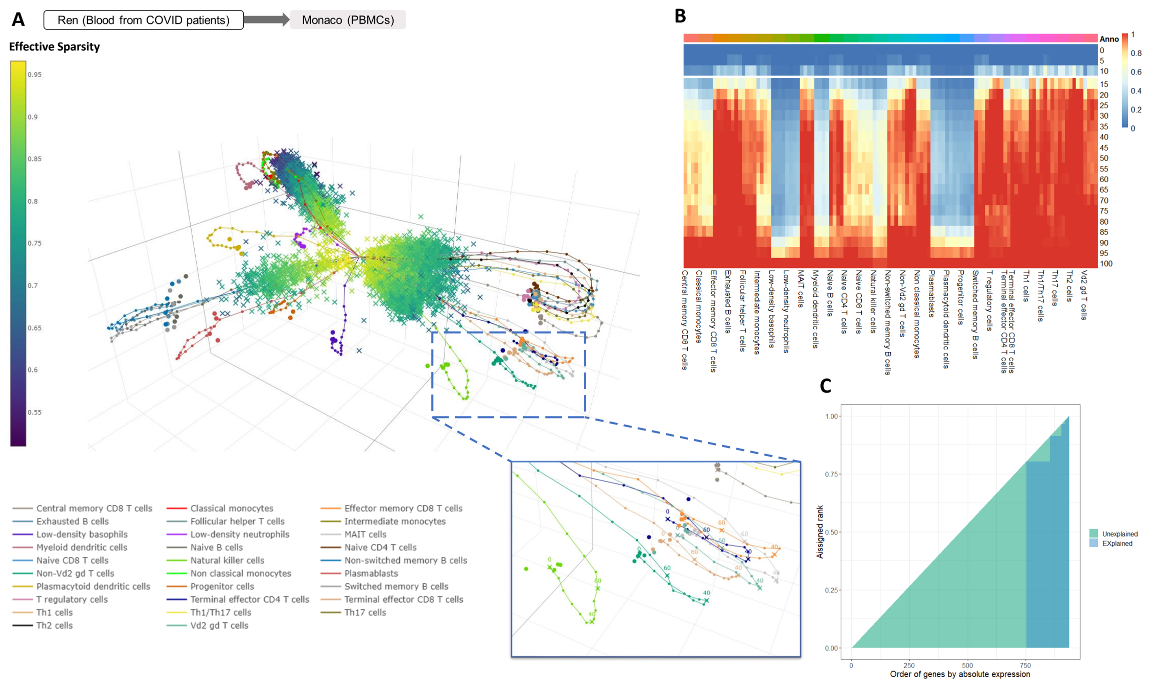

Figure 1. Impact of sparsity on projection. **(A)** Non aggregated query cells from Ren et al. [22] projected on the Monaco et al. [21] atlas, and colored by effective sparsity rate. We down-sampled bulk samples in the atlas for sparsity rates ranging from 0 to 100 percent with a step of 5 to simulate pseudo-single cells. Projection of these pseudo-single cells for each sparsity rate are shown as cluster centroids trajectories'. The sparsity of query single cells matched the sparsity of the pseudo-single cell clusters around which query cells were projected, suggesting that deviation of query clusters from the atlas clusters is due to sparsity, rather than biology. **(B)** Minimum inter-cluster distance to minimum intra-cluster distance ratio calculated on the Monaco et al. [21] pseudo-single cells simulated for each at each sparsity rate. The cluster of a pseudo-single cell is defined by its corresponding atlas cluster whereby down-sampling has not been performed. The change of distance ratio for each cluster matches the simulated cluster trajectories. When sparsity is less than 15 percent, 90 percent of pseudo-single cells retained a distance ratio smaller than 0.5. Hence, querying single cells on the Monaco et al. [21] atlas may not be largely impacted when sparsity of the query data is less than 15 percent. **(C)** Area plot showing the rank expression of genes within a query cell. On the x-axis, genes are ordered by their absolute expression rank. The blue region depicts the observed ranking. If all genes are distinctly expressed with no ties, the blue region should fill the whole triangle. The green region represents loss of variation in the cell due to ties in expression. Gene rank expression in a cell is not only influenced by sparsity, but also ties in gene expression.

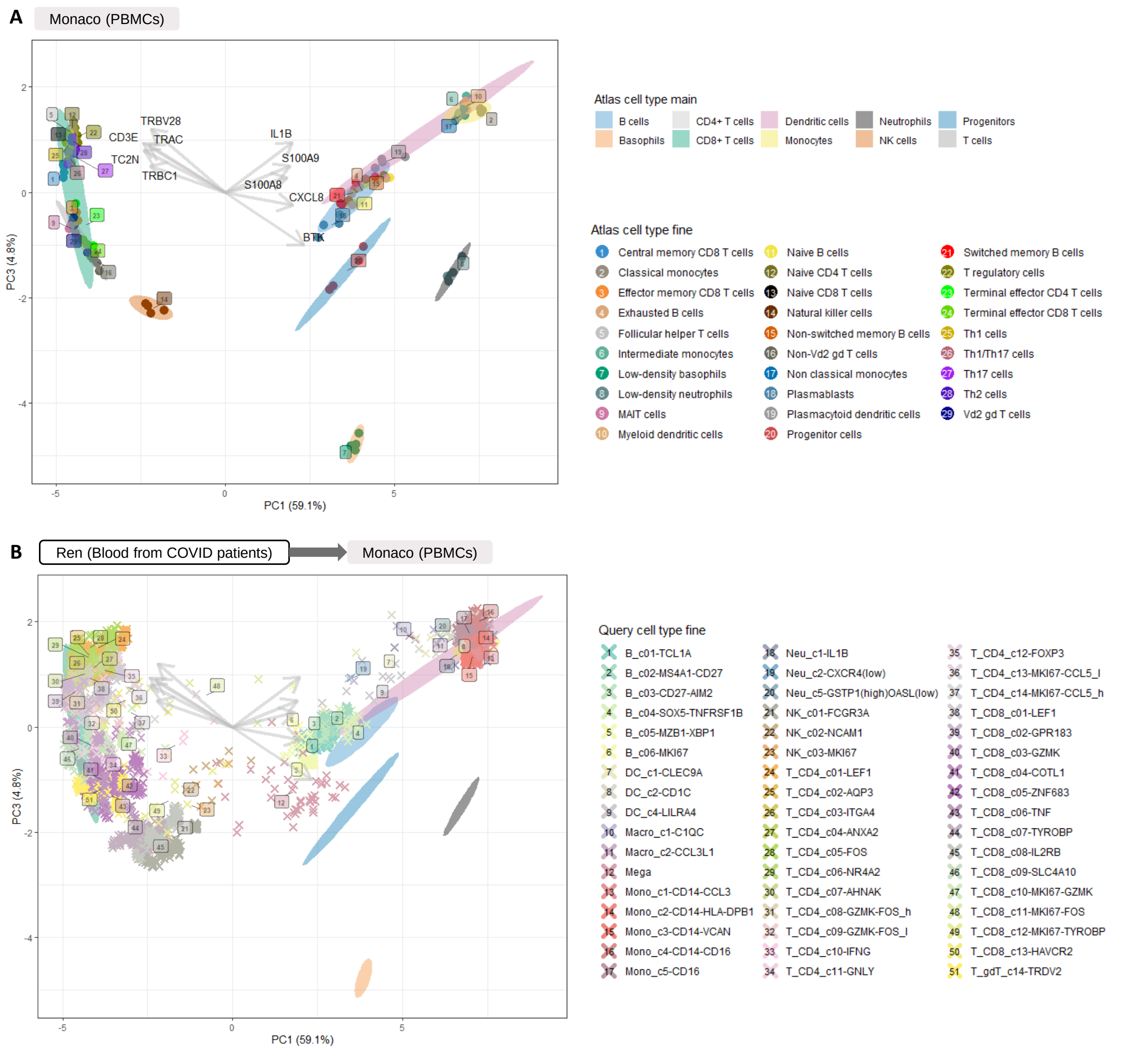

Figure 2. Querying Ren et al. [22] on the Monaco et al. [21] atlas. **(A)** Samples from the reference atlas, annotated by the atlas fine cell type labels. **(B)** Query pseudo-bulk projected on the reference atlas, and annotated by the query fine cell type labels.

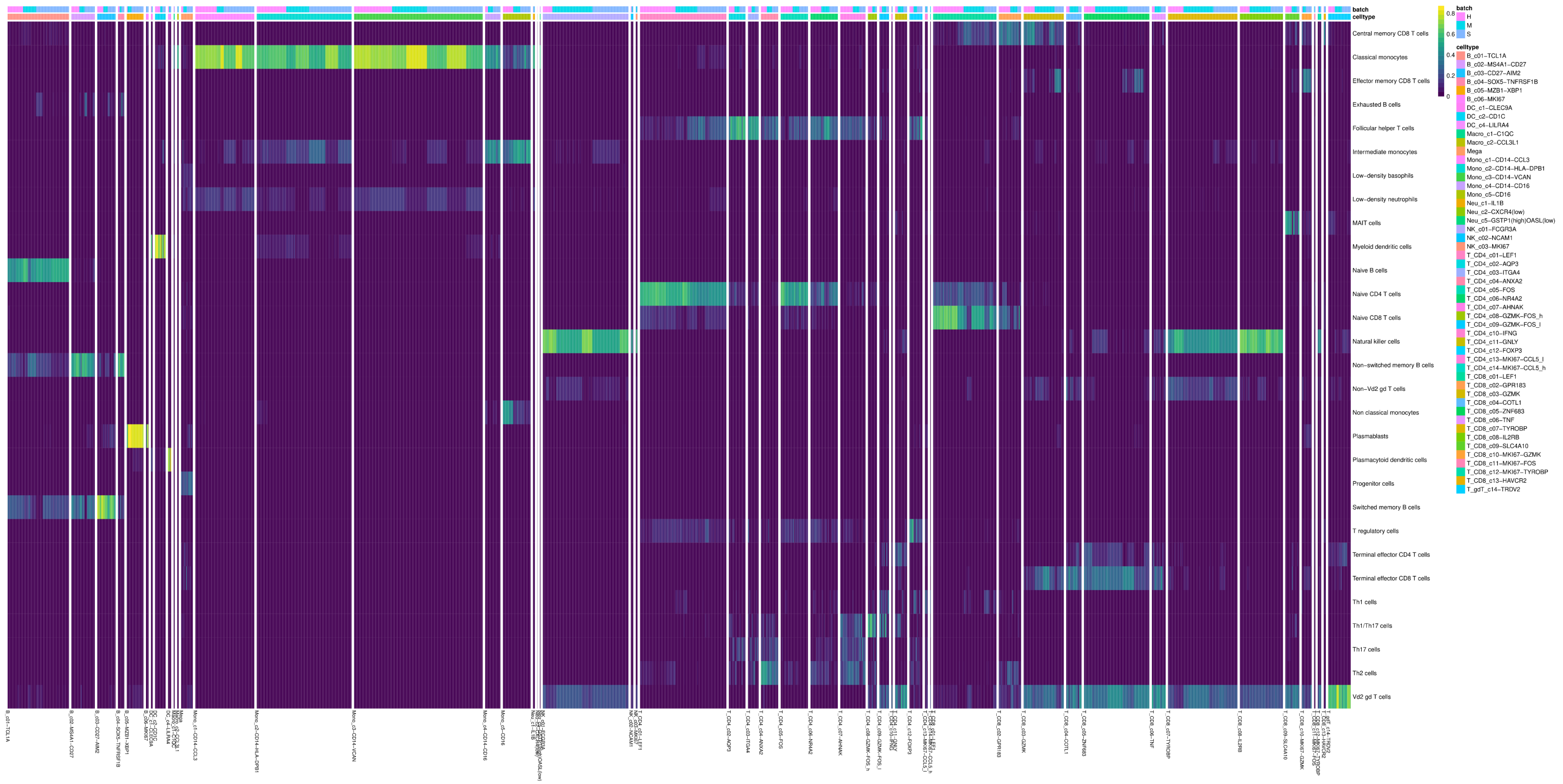

Figure 3. Improved Capybara cell score heatmap predicted on pseudo-bulk samples from Ren et al. [22] projected onto the reference Monaco et al. [21] atlas.

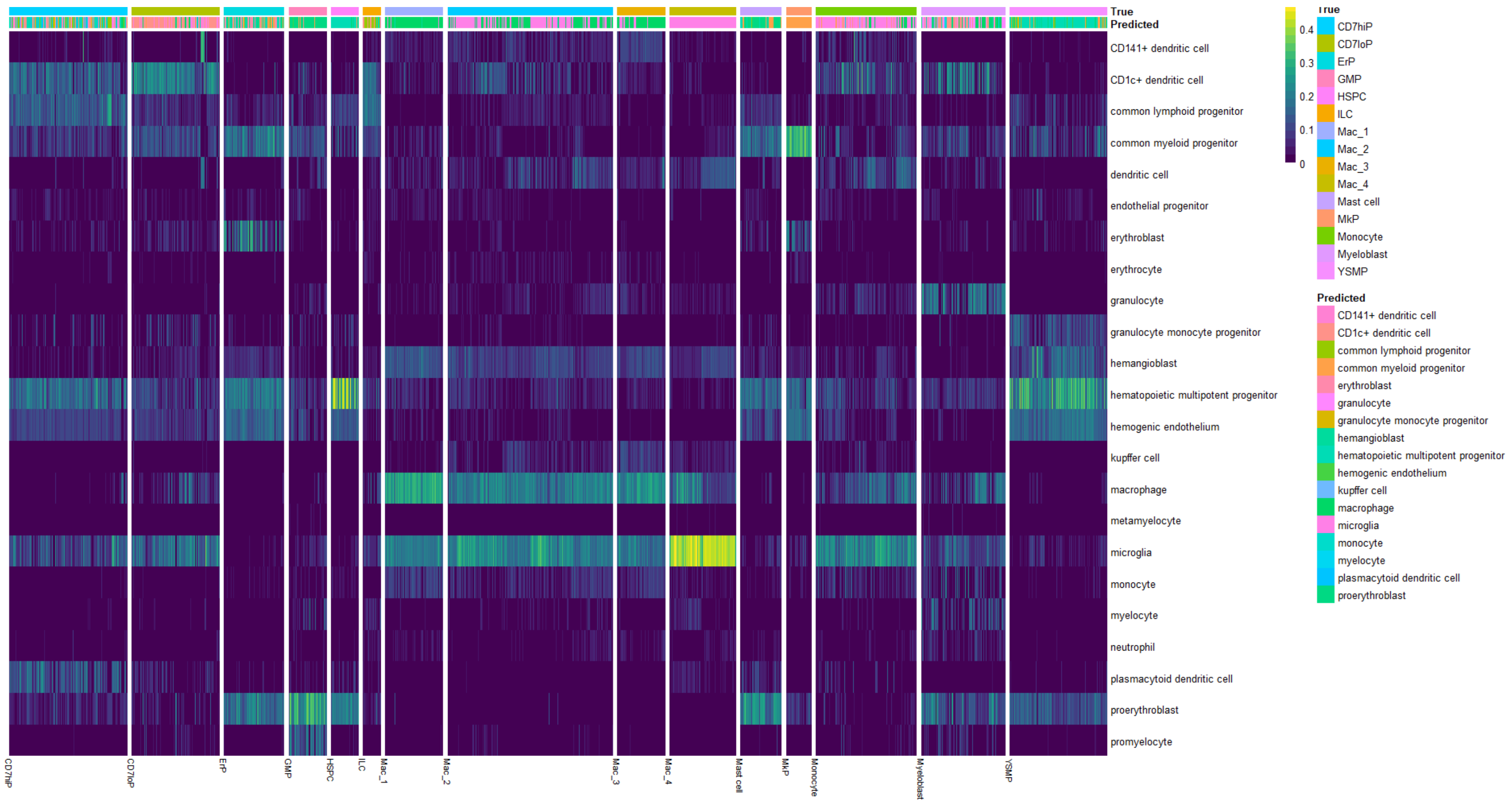

Figure 4. Improved Capybara cell score heatmap predicted on Sincast imputed cells from Bian et al. [25] projected onto the reference Rajab et al. [24] atlas.

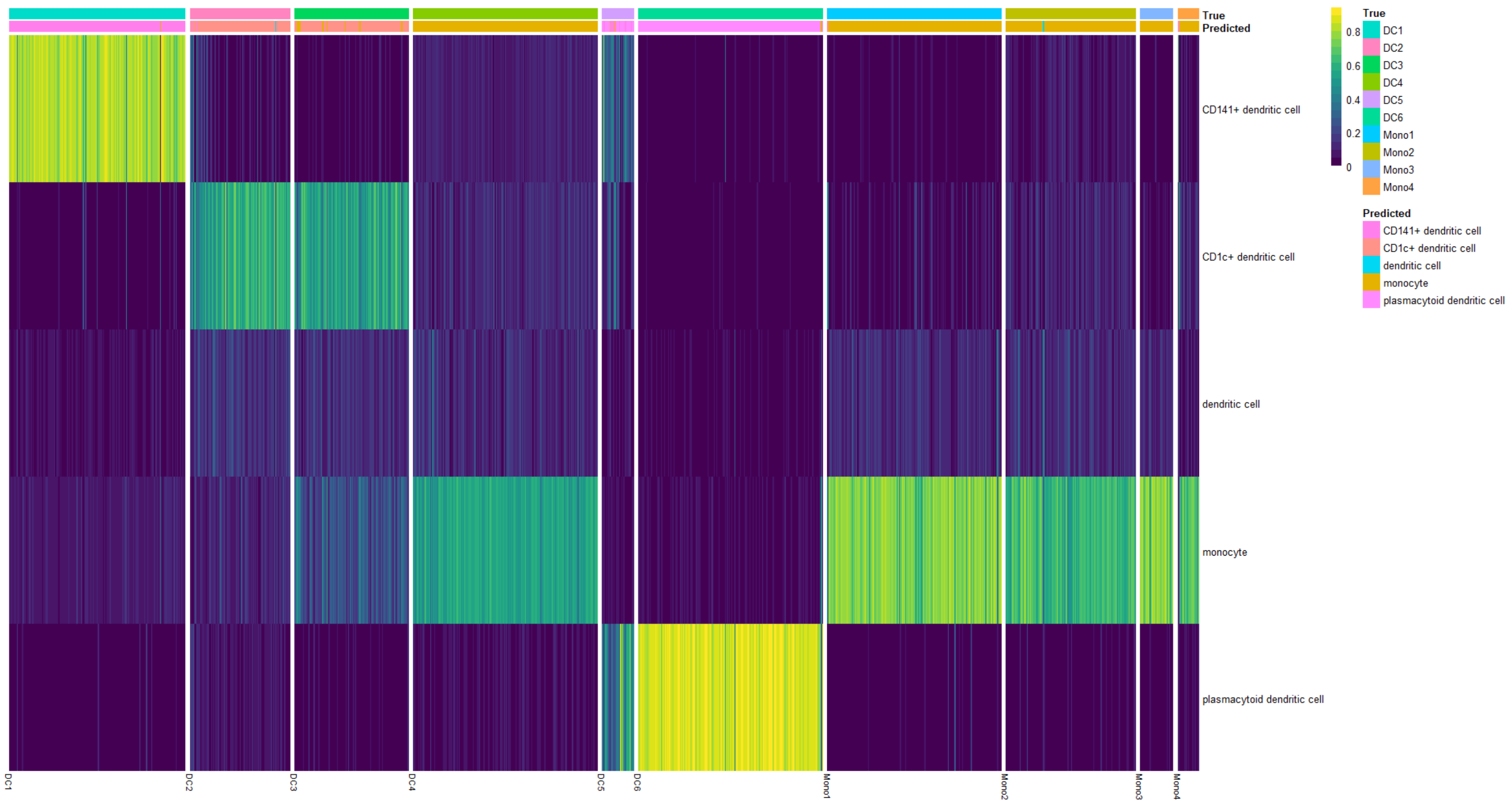

Figure 5. Improved Capybara cell score heatmap predicted on Sincast imputed cells from Villani et al. [23] projected onto the reference Monocyte-Dendritic cell subset of the Rajab et al. [24] atlas.

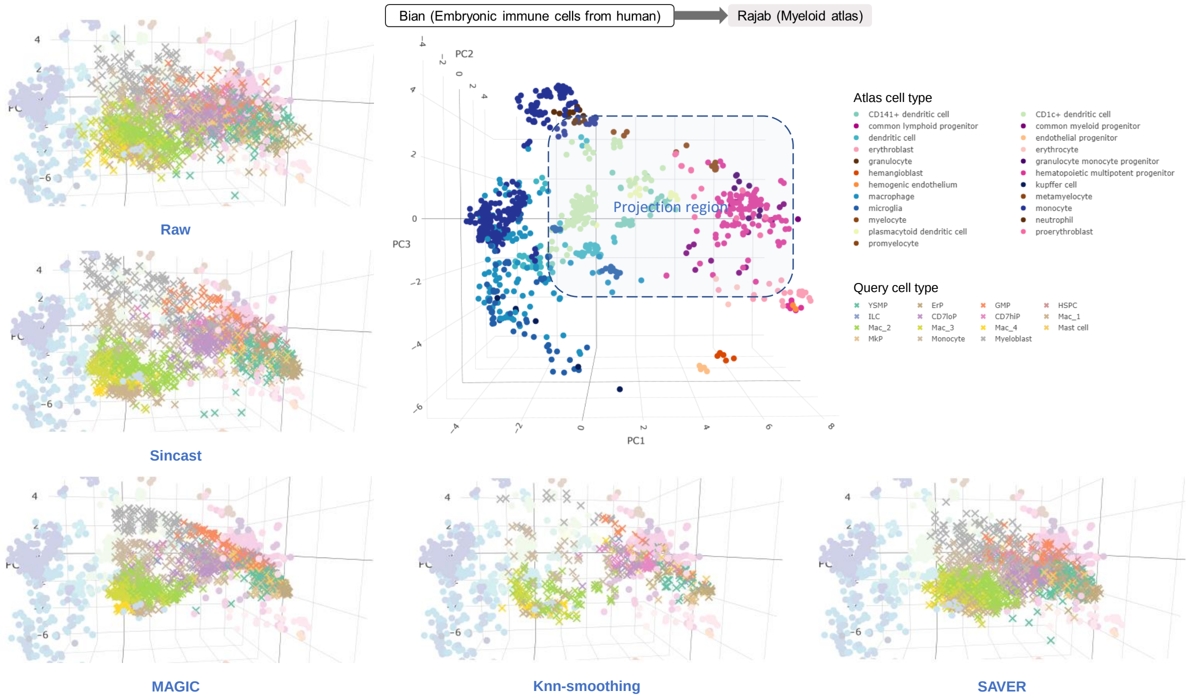

Figure 6. Projection of cells from Bian et al. [25] on the Rajab et al. [24] atlas when cell imputation is performed. Different imputation methods led to different data structures in the query due to their algorithmic assumptions. For example, the distribution of MAGIC and SAVER imputed data were shrunk towards the middle of the atlas, potentially indicating improper post-imputation data scaling. The distribution of knn-smoothing imputed data was scattered as a result of aggregating cells locally at each cell's neighborhood.

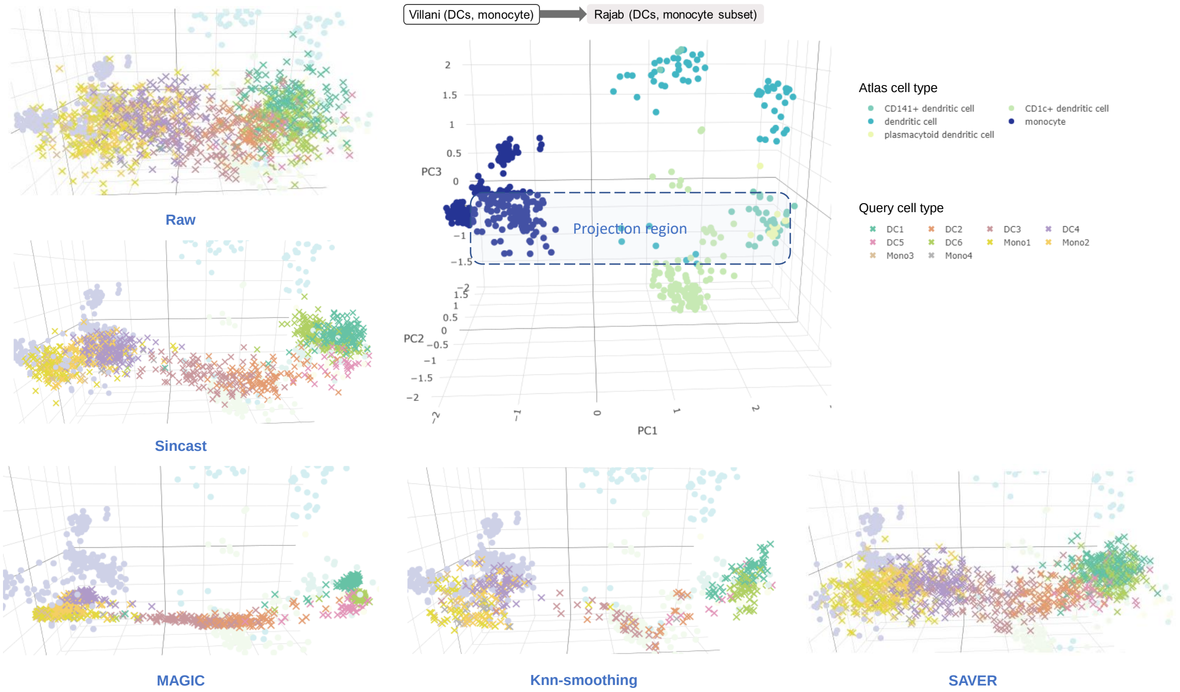

Figure 7. Projection of cells from Villani et al. [23] imputed by different imputation methods, on the Monocyte-Dendritic cell subset of the Rajab et al. [24] atlas.

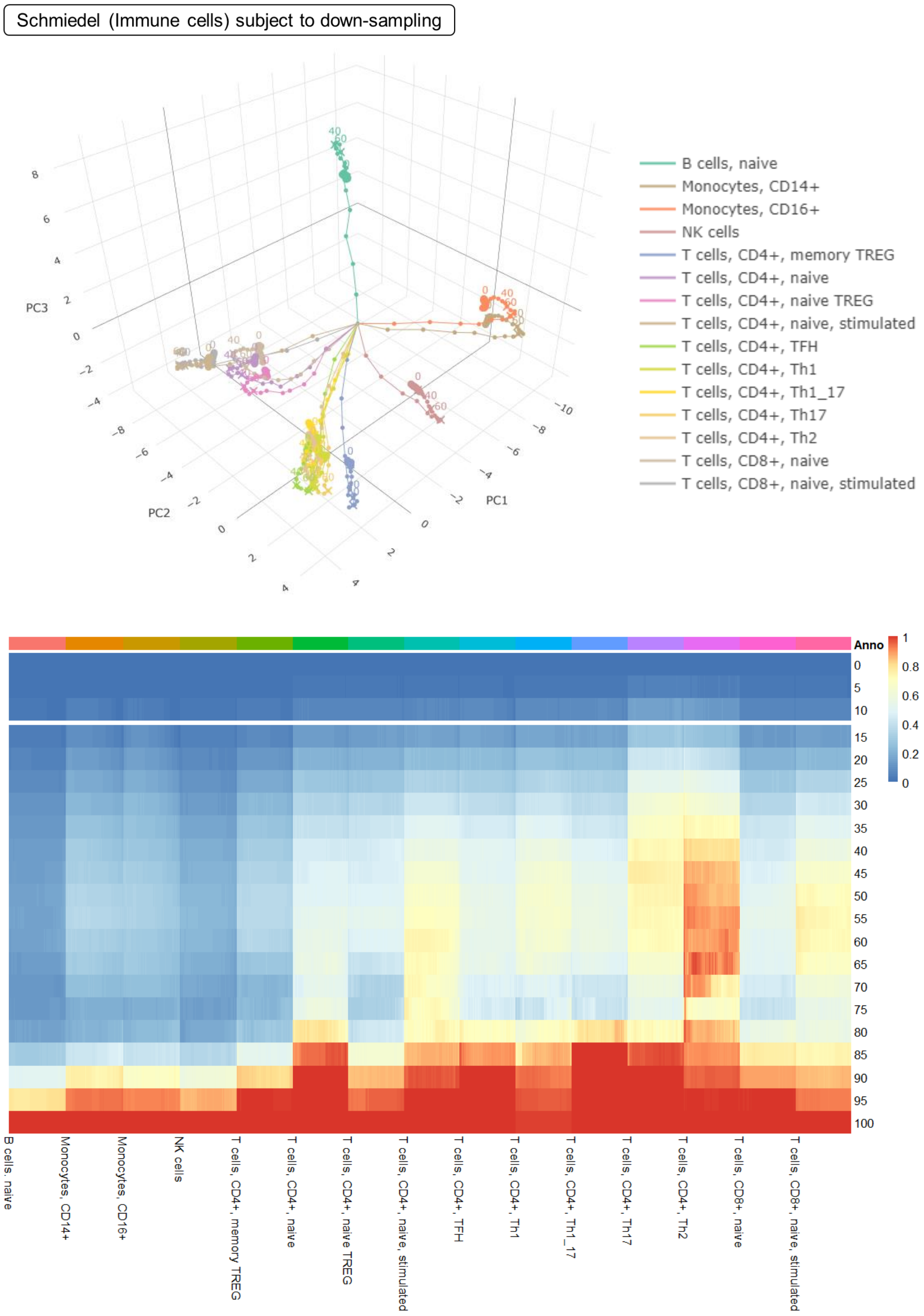

Figure 8. Impact of sparsity on projecting cells to the Schmiedel et al. [11] reference atlas. Similar to Figure 1 we down-sampled bulk samples in the atlas at sparsity rates ranging from 0 to 100 percent. The top panel shows the atlas and its simulated cluster trajectories. The bottom panel shows each simulated cell's minimum inter-cluster distance to minimum intra-cluster distance ratio. When sparsity is less than 15 percent, 90 percent of pseudo-single cells retained a distance ratio smaller than 0.5. Hence, querying single cells on the Schmiedel et al. [11] atlas may not be impacted when the sparsity of the query data is less than 15 percent.

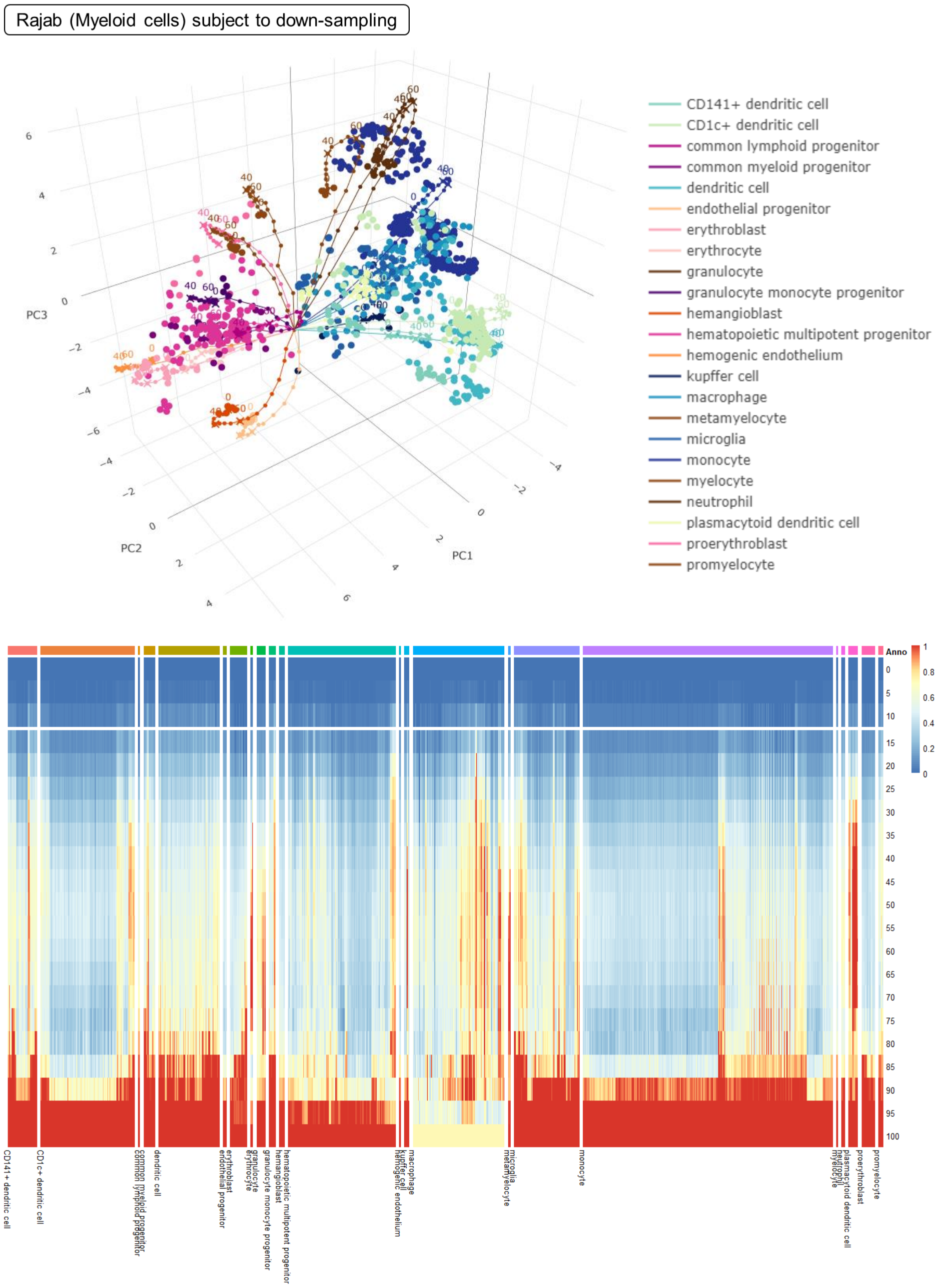

Figure 9. Impact of sparsity on projecting cells to the Rajab et al. [24] reference atlas. Similar to Figures 1 and 8 we conclude that querying single cells on the Rajab et al. [24] atlas may not be impacted when the sparsity of the query data is less than 15 percent.

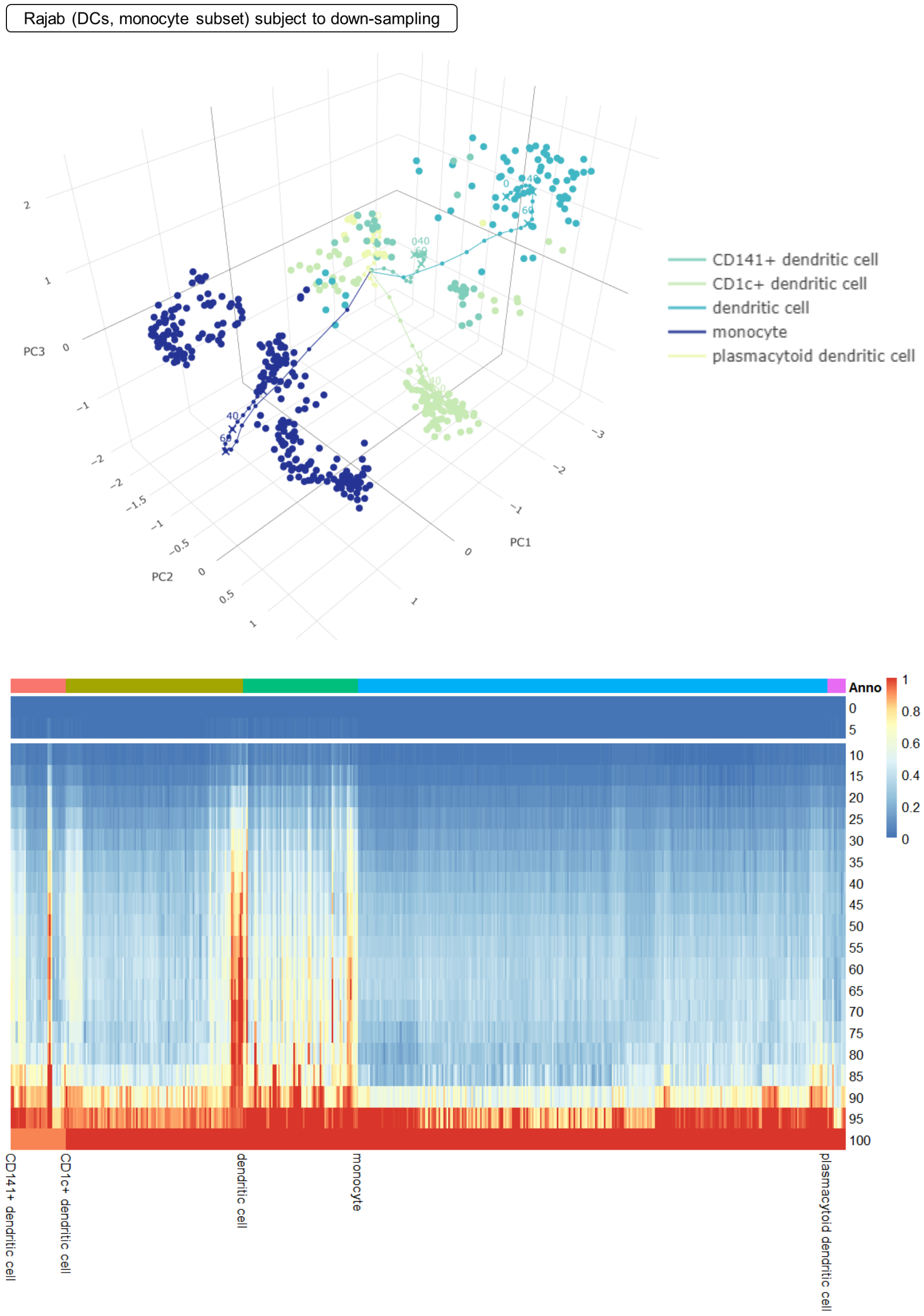

Figure 10. Impact of sparsity on projecting cells to the Monocyte-Dendritic cell subset of Rajab et al. [24] atlas with an analysis similar to Figures 1, 8 and 9. When sparsity is less than 10 percent, 90 percent of pseudo-single cells retained distance ratio smaller than 0.5. We conclude that querying single cells on the Rajab et al. [24] atlas may not be impacted when the sparsity of the query data is less than 10 percent.

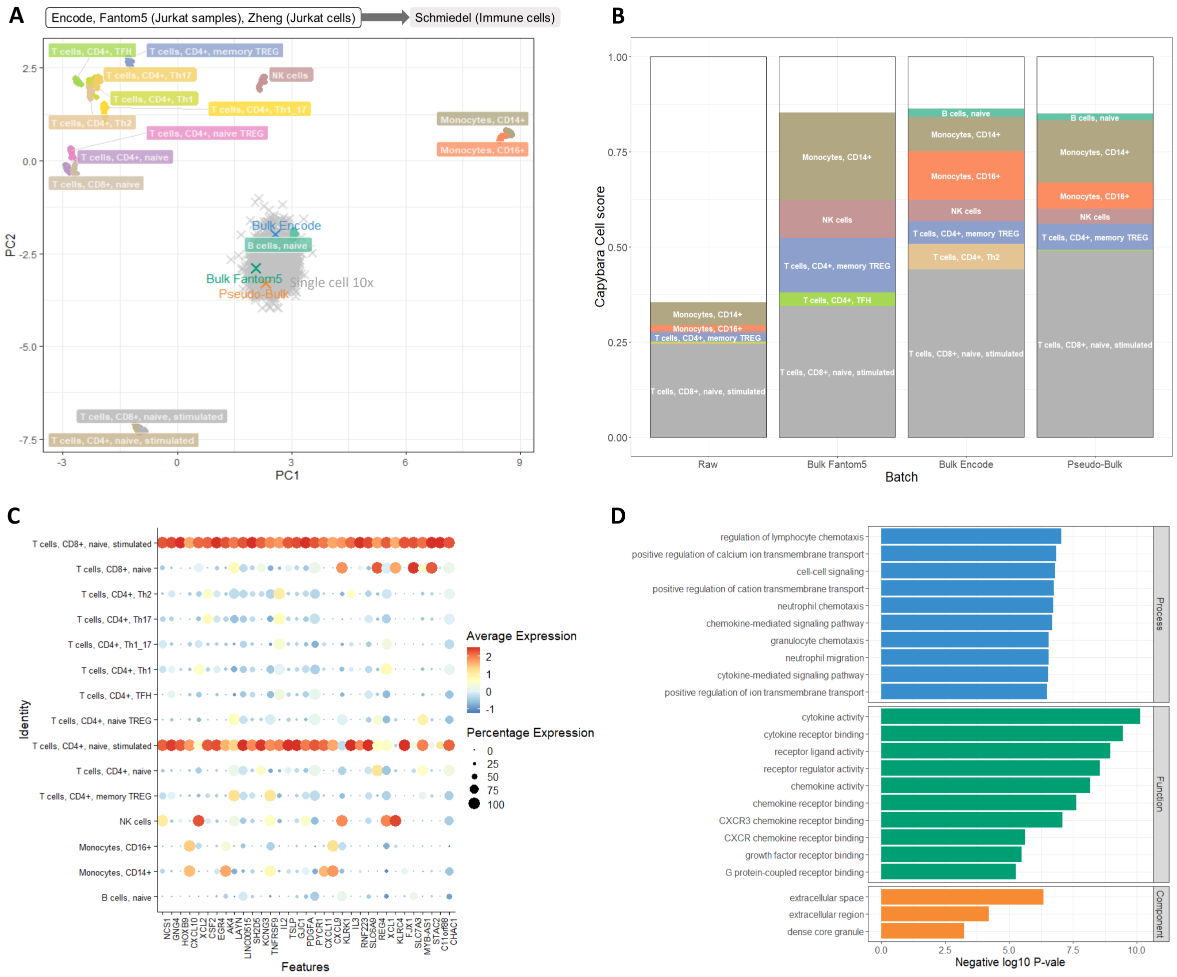

Figure 11. Profiling Jurkat T cell identity on the Schmiedel et al. [11] atlas. **(A)** On the atlas PC1 and PC3, projection of Jurkat single cell line from Zheng et al. [12], Bulk Jurkat samples from Fantom5 project [13] and Encode project [14], and pseudo-bulk samples aggregated by Jurkat single cells. **(B)** Averaged capybara cell score predicted on the query projections in (A). Heights of the color bars represent the scores query obtained on the different reference cell types. Pseudo-bulk and real bulk samples of Jurkat cell line shared similar transcriptional identity as revealed by their cell score composition. **(C)** We performed differential expression test on the stimulated naive CD8 T cells (CD8-TA) of the atlases versus other atlas samples. Expression of top 36 differentially expressed genes in the atlas samples shown as dotplot. CD8-TA marker gene set overlaps with known markers of Jurkat T cells. **(D)** Gene Ontology enrichment analysis performed on genes from (C).

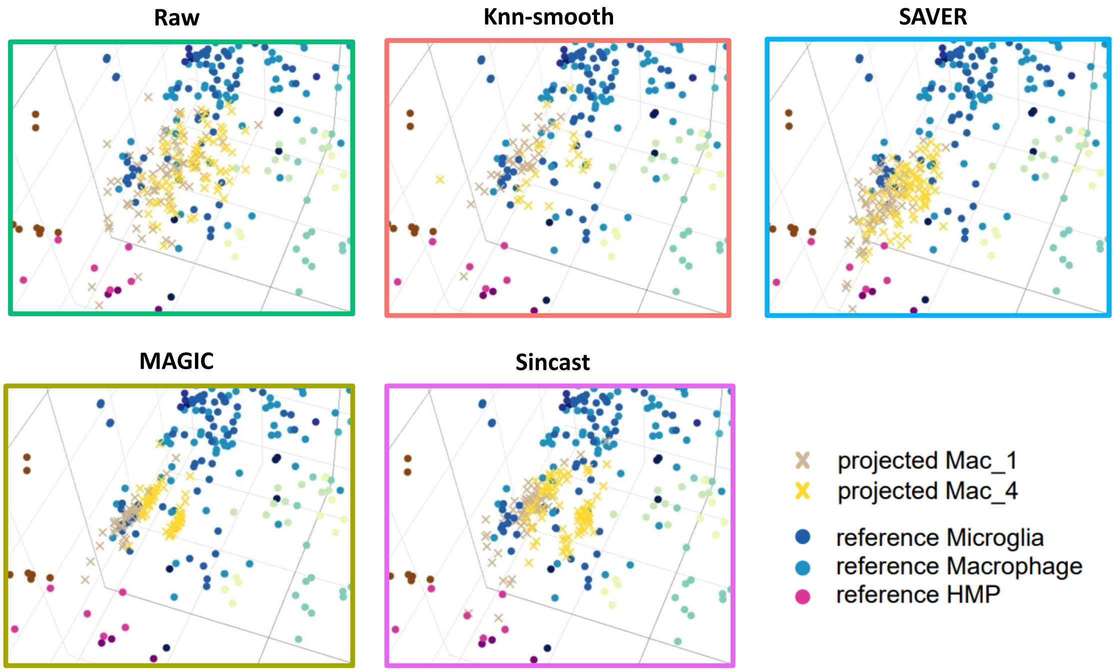

Figure 12. Mac_1 and Mac_4 query cells from Bian et al. [25] were imputed using different methods prior to projection onto the Rajab et al. [24] atlas. Close-up on the relevant area of PCA space is shown. Only MAGIC and Sincast imputation methods result in separation of these two clusters when projected, consistent with observations made by Bian et al. [25].

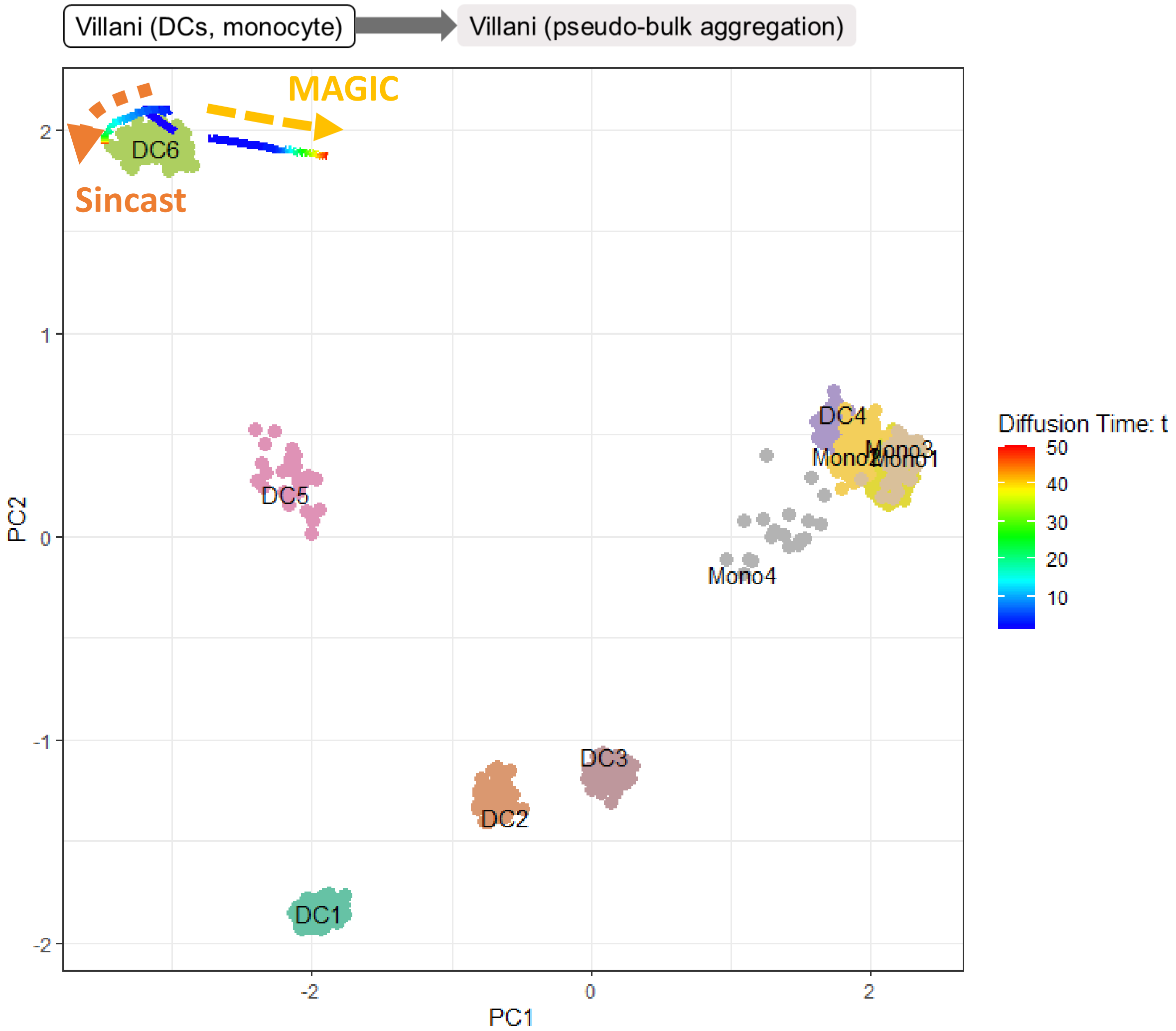

Figure 13. Projection of Sincast and the MAGIC imputed DC6 (pDC) cell population from Villani et al. [23] onto the monocyte-DC subset of the Rajab et al. [24] atlas. Imputation was performed on the 20 DC6 and 287 monocyte subsets of the Villani et al. [23] data (the number of DC6 is increased from 10 to 20 compared to Figure 4B). The imputation neighborhood size is set to 15, which is smaller than the actual size of DC6 population included in the test data. Projection centroids of the imputed DC6 population with increasing diffusion time t are shown. The shift of MAGIC imputed DC6 population towards monocyte identity was not as strong as in Figure 4B, suggesting strong impact of tuning on MAGIC imputation.

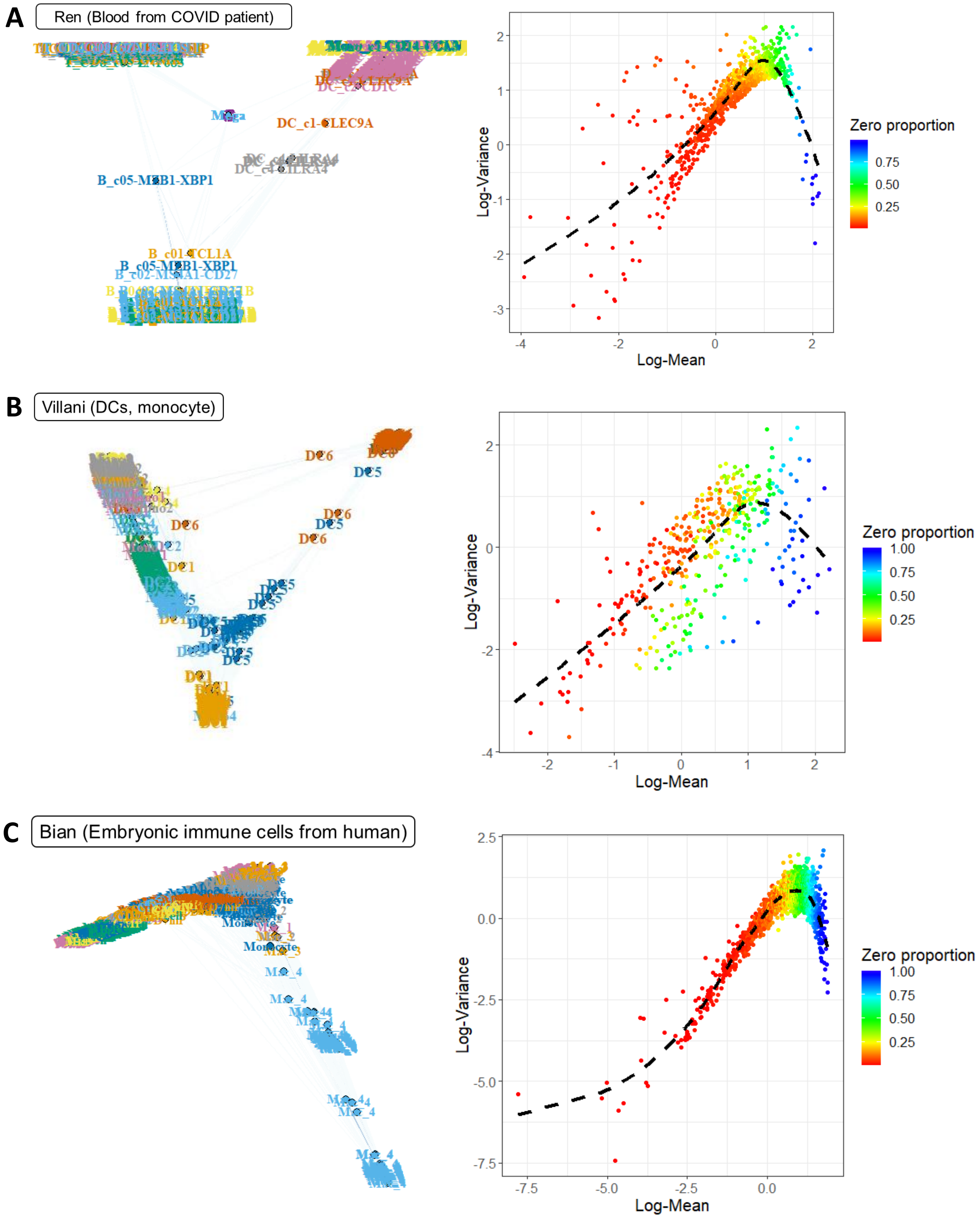

Figure 14. (Caption continued in the next page.)

Figure 14. Diagnostic plots for Sincast imputation and post-imputation data scaling, made independently for **(A)** Ren et al. [22], **(B)** Villani et al. [23] and **(C)** Villani et al. [23] data. For each query data, the top panel shows the diffusion embedding learnt by eigen decomposition of the diffusion operator used for Sincast data imputation. Cells in the embedding are connected by weighted lines (edges) representing affinities. The Sincast diffusion embedding gives some intuition on how the query cells are connected and hence impute each other in the graph defined by Sincast. For example, in (A) we can observe that the graph constructed was much sparser than in (B) and (C), informing that cells of different components in graph such as myeloid cells and lymphoid cells rarely impute each other. The bottom panel shows the log-gene mean and variance relationship representing global gene dispersion trend in the imputed data. Dashed black line represents generalized additive model fitting on the trend. Different data showed similar trend, suggesting that there are consistent dispersion trends to be estimated in scRNA-seq data. We scaled the imputed data according to their trend estimation.

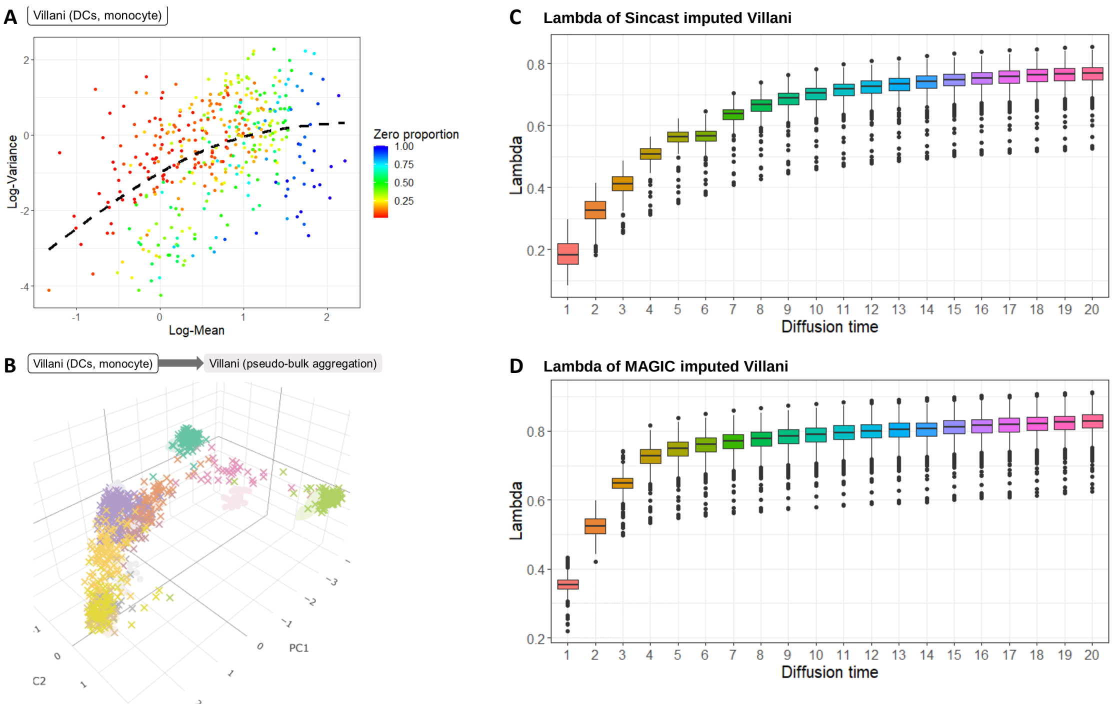

Figure 15. Sincast post-imputation data scaling on MAGIC imputed Villani et al. [23] data. **(A)** Log-gene mean and variance relationship estimated on MAGIC imputed data (similar to Figure 14B, top panel for Sincast), showing that MAGIC and Sincast imputed data share similar dispersion trends. **(B)** Projection of MAGIC imputed-Sincast scaled data onto a pseudo-bulk version of the Villani et al. [23] data. The loss of local and global variation after imputation was successfully recovered by Sincast data scaling (Compared to Figure 4A, yellow panel for MAGIC). **(C)** Distribution of scaling strength lambda for each imputed cell with Sincast. When lambda increases, the imputed cells tend to shrink back to the original, unimputed observations. As imputation strength increases (diffusion time t), lambda also increased in response. **(D)** Similar to (C) but for MAGIC imputed data. Compared to Sincast imputation, the lambda's increase was more sharp as diffusion time increased, suggesting that MAGIC has a stronger imputation strength than Sincast, potentially leading to over-smoothing.

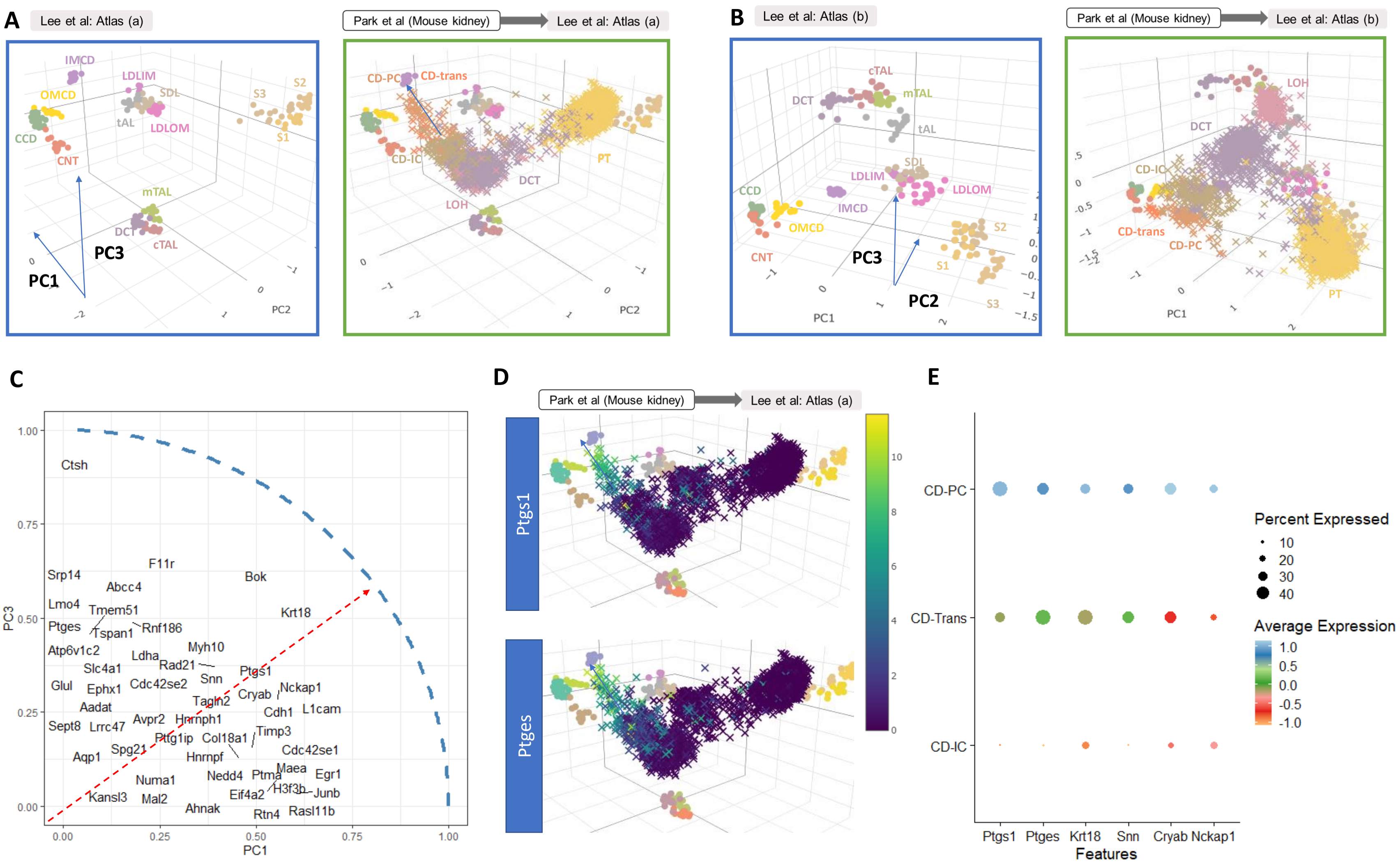

Figure 16. (Caption continued in the next page.)

Figure 16. Projection of mouse kidney single cells from Park et al. [16] onto the reference atlas of micro-dissected rat kidney tubules segments from Lee et al. [15]. **(A)** Sincast projection with query cells imputation onto the atlas (a) based on cell type discriminant genes. Atlas with text indicating the reference cell types (left, circles) or the query cell types (right, crosses). Query cells are projected in the right directions of the atlas, in agreement with their matching bulk reference samples. However, this projection underfits the atlas, highlighting differences between species. **(B)** Sincast projection with query cells imputation onto the atlas (b) based on query cell type markers provided by Park et al. [16]. Atlas with text indicating the reference cell types (left, circles) or the query cell types (right, crosses). Compared to the projection in (A), the projection in (B) fits the atlas samples better, but we also observed some overfitting as the query PT cells exceeds the distribution range of the atlas. **(C)** Loadings plot on Principal Components 1 and 3 from the atlas. Red dashed line indicated the IC to PC transition. Genes close to the red line and the edge of the blue circle are more likely to drive the transition. **(D)** Log-expression of Ptgs1 and Ptges gene expression in (A), showing similar expression patterns. **(E)** The dotplot shows the expression of genes close to the red line and the edge of the blue circle in (C). The increasing expression of these genes from IC to PC cell suggests their potential association with transition.
Reference cell types: S1 = first segment of the proximal tubule; S2 = second segment of the proximal tubule; S3 = third segment of the proximal tubule; SDL = short descending limb of the loop of Henle; LDLOM = long descending limb of the loop of Henle in the outer medulla; LDLIM = long descending limb of the loop of Henle in the inner medulla; tAL = thin ascending limb of the loop of Henle; mTAL = medullary thick ascending limb of the loop of Henle; cTAL = cortical thick ascending limb of the loop of Henle; DCT = distal convoluted tubule; CNT = connecting tubule; CCD = cortical collecting duct; OMCD = outer medullary collecting duct; IMCD = inner medullary collecting duct. **Query cell types**: PT = proximal tubule, LOH = ascending loop of Henle, DCT = distal convoluted tubule, CD-PC = collecting duct principal cell, CD-IC = CD intercalated cell, CD-Trans = CD transitional cell.

References

1. Benjamin M Bolstad, Rafael A Irizarry, Magnus Åstrand, and Terence P. Speed. A comparison of normalization methods for high density oligonucleotide array data based on variance and bias. *Bioinformatics*, 19(2):185–193, 2003.

2. Paul W Angel, Nadia Rajab, Yidi Deng, Chris M Pacheco, Tyrone Chen, Kim-Anh Lê Cao, Jarny Choi, and Christine A Wells. A simple, scalable approach to building a cross-platform transcriptome atlas. *PLoS computational biology*, 16(9):e1008219, 2020.

3. Kailin Tang, Xuejie Ji, Mengdi Zhou, Zeliang Deng, Yuwei Huang, Genhui Zheng, and Zhiwei Cao. Rank-in: enabling integrative analysis across microarray and rna-seq for cancer. *Nucleic Acids Research*, 2021.

4. Guang-Hui Fu, Yuan-Jiao Wu, Min-Jie Zong, and Jianxin Pan. Hellinger distance-based stable sparse feature selection for high-dimensional class-imbalanced data. *BMC bioinformatics*, 21(1):1–14, 2020.

5. David A Cieslak, T Ryan Hoens, Nitesh V Chawla, and W Philip Kegelmeyer. Hellinger distance decision trees are robust and skew-insensitive. *Data Mining and Knowledge Discovery*, 24(1):136–158, 2012.

6. Simon N Wood. Fast stable restricted maximum likelihood and marginal likelihood estimation of semiparametric generalized linear models. *Journal of the Royal Statistical Society: Series B (Statistical Methodology)*, 73(1):3–36, 2011.

7. Peter J Rousseeuw. Silhouettes: a graphical aid to the interpretation and validation of cluster analysis. *Journal of computational and applied mathematics*, 20:53–65, 1987.

8. Lawrence Hubert and Phipps Arabie. Comparing partitions. *Journal of classification*, 2(1):193–218, 1985.

9. Alboukadel Kassambara and Fabian Mundt. Package 'factoextra'. *Extract and visualize the results of multivariate data analyses*, 76, 2017.

10. Christian Hennig. *fpc: Flexible Procedures for Clustering*, 2020. URL https://CRAN.R-project.org/package=fpc. R package version 2.2-5.

11. Benjamin J Schmiedel, Divya Singh, Ariel Madrigal, Alan G Valdovino-Gonzalez, Brandie M White, Jose Zapardiel-Gonzalo, Brendan Ha, Gokmen Altay, Jason A Greenbaum, Graham McVicker, et al. Impact of genetic polymorphisms on human immune cell gene expression. *Cell*, 175(6):1701–1715, 2018.

12. Grace XY Zheng, Jessica M Terry, Phillip Belgrader, Paul Ryvkin, Zachary W Bent, Ryan Wilson, Solongo B Ziraldo, Tobias D Wheeler, Geoff P McDermott, Junjie Zhu, et al. Massively parallel digital transcriptional profiling of single cells. *Nature communications*, 8(1):1–12, 2017.

13. Marina Lizio, Jayson Harshbarger, Hisashi Shimoji, Jessica Severin, Takeya Kasukawa, Serkan Sahin, Imad Abugessaisa, Shiro Fukuda, Fumi Hori, Sachi Ishikawa-Kato, et al. Gateways to the fantom5 promoter level mammalian expression atlas. *Genome biology*, 16(1):1–14, 2015.

14. Carrie A Davis, Benjamin C Hitz, Cricket A Sloan, Esther T Chan, Jean M Davidson, Idan Gabdank, Jason A Hilton, Kriti Jain, Ulugbek K Baymuradov, Aditi K Narayanan, et al. The encyclopedia of dna elements (encode): data portal update. *Nucleic acids research*, 46(D1):D794–D801, 2018.

15. Jae Wook Lee, Chung-Lin Chou, and Mark A Knepper. Deep sequencing in microdissected renal tubules identifies nephron segment–specific transcriptomes. *Journal of the American Society of Nephrology*, 26(11):2669–2677, 2015.

16. Jihwan Park, Rojesh Shrestha, Chengxiang Qiu, Ayano Kondo, Shizheng Huang, Max Werth, Mingyao Li, Jonathan Barasch, and Katalin Suszták. Single-cell transcriptomics of the mouse kidney reveals potential cellular targets of kidney disease. *Science*, 360(6390):758–763, 2018.

17. David Pearce, Rama Soundararajan, Christiane Trimpert, Ossama B Kashlan, Peter MT Deen, and Donald E Kohan. Collecting duct principal cell transport processes and their regulation. *Clinical journal of the American Society of Nephrology*, 10(1):135–146, 2015.

18. Yuyuan Li, Yuanyi Wei, Feng Zheng, Youfei Guan, and Xiaoyan Zhang. Prostaglandin e2 in the regulation of water transport in renal collecting ducts. *International Journal of Molecular Sciences*, 18(12):2539, 2017.

19. Biao Chen, Ximing Xu, Dan-dan Lin, Xin Chen, Yang-tao Xu, Xin Liu, and Wei-guo Dong. Krt18 modulates alternative splicing of genes involved in proliferation and apoptosis processes in both gastric cancer cells and clinical samples. *Frontiers in genetics*, 12, 2021.

20. Renee Rao, Vivek Bhalla, and Núria M Pastor-Soler. Intercalated cells of the kidney collecting duct in kidney physiology. In *Seminars in nephrology*, volume 39, pages 353–367. Elsevier, 2019.

21. Gianni Monaco, Bernett Lee, Weili Xu, Seri Mustafah, You Yi Hwang, Christophe Carre, Nicolas Burdin, Lucian Visan, Michele Ceccarelli, Michael Poidinger, et al. Rna-seq signatures normalized by mrna abundance allow absolute deconvolution of human immune cell types. *Cell reports*, 26(6):1627–1640, 2019.

22. Xianwen Ren, Wen Wen, Xiaoying Fan, Wenhong Hou, Bin Su, Pengfei Cai, Jiesheng Li, Yang Liu, Fei Tang, Fan Zhang, et al. Covid-19 immune features revealed by a large-scale single-cell transcriptome atlas. *Cell*, 184(7):1895–1913, 2021.

23. Alexandra-Chloé Villani, Rahul Satija, Gary Reynolds, Siranush Sarkizova, Karthik Shekhar, James Fletcher, Morgane Griesbeck, Andrew Butler, Shiwei Zheng, Suzan Lazo, et al. Single-cell rna-seq reveals new types of human blood dendritic cells, monocytes, and progenitors. *Science*, 356(6335), 2017.

24. Nadia Rajab, Paul W Angel, Yidi Deng, Jennifer Gu, Vanta Jameson, Mariola Kurowska-Stolarska, Simon Milling, Chris M Pacheco, Matt Rutar, Andrew L Laslett, et al. An integrated analysis of human myeloid cells identifies gaps in in vitro models of in vivo biology. *Stem cell reports*, 16(6):1629–1643, 2021.

25. Zhilei Bian, Yandong Gong, Tao Huang, Christopher ZW Lee, Lihong Bian, Zhijie Bai, Hui Shi, Yang Zeng, Chen Liu, Jian He, et al. Deciphering human macrophage development at single-cell resolution. *Nature*, pages 1–6, 2020.
